# Comprehensive evolutionary analysis of the *TCP* gene family: Further insights for its origin, expansion, and diversification

**DOI:** 10.3389/fpls.2022.994567

**Published:** 2022-09-02

**Authors:** Jun-Li Wang, Hong-Wei Wang, Ya-Nan Cao, Sheng-Long Kan, Yan-Yan Liu

**Affiliations:** ^1^College of Plant Protection, Henan Agricultural University, Zhengzhou, China; ^2^Guangdong Laboratory of Lingnan Modern Agriculture, Genome Analysis Laboratory of the Ministry of Agriculture and Rural Affairs, Agricultural Genomics Institute at Shenzhen, Chinese Academy of Agricultural Sciences, Shenzhen, China

**Keywords:** TCP, CYC, gene family expansion, gene duplication, green plants

## Abstract

TCP proteins are plant-specific transcription factors, which are involved in a broad range of physiological processes of plant growth and development. However, the origin and evolutionary history of this gene family is not fully resolved. Here, we present a genome-wide survey of *TCP* genes in 59 species (including 42 genomes and 17 transcriptomes) covering all main lineages of green plants, and reconstruct the evolutionary history of this gene family. Our results suggested that the origin of *TCP* genes predated the emergence of land plants, possibly in the common ancestor of Phragmoplastophyta. The *TCP* gene family gradually experienced a continuous expansion and grew from a few members in algae, moss and lycophytes to dozens, and sometimes over 50 members in angiosperms. Phylogenetic analysis indicated that at least four subclades (Class I and three subclades of Class II) have been occurred in the ancestor of spermatophyte (seed plant). Both dispersed duplication and segmental duplication or whole-genome duplication (WGD) contributed significantly to the expansion of the *TCP* gene family over the course of evolution. Our findings provide a comprehensive evolutionary analysis of the *TCP* gene family and highlight the importance of gene duplications in the evolution of this plant-specific transcription factors.

## Introduction

Transcription factors (TFs) constitute major components of the genetic basis for phenotypic evolution (Wray et al., 2003). Variations of TF, such as the expansion and diversification, play central roles in the evolution of some key innovation of green plants, for example, vascular tissues, megaphylls, roots, reproductive cones, or flowers (Floyd and Bowman, 2007; Lai et al., 2020). With the unprecedented pace of sequencing of genomes and transcriptomes, the repertoire of TFs from a wide variety of green plant species (ranging from algae to angiosperms) will be better characterized. The advent of massive sequence information not only helps to identify the TF family members but also allows a comprehensive investigation of how TF families have expanded and how the expansion has provided members involved in multiple specific innovations.

TCP proteins (TCPs) are plant-specific TFs, which was initially identified and named after *TEOSINTE BRANCHED 1* (*TB1*) in *Zea mays* (Doebley et al., 1997), *CYCLOIDEA* (*CYC*) in *Antirrhinum majus* (Luo et al., 1996), *PROLIFERATING CELL FACTORS 1* and *2* (*PCF1* and *PCF2*) in *Oryza sativa* (Kosugi and Ohashi, 1997). All known TCPs are characterized by a 59-amino acid basic helix-loop-helix (bHLH) domain known as TCP domain (Cubas et al., 1999). Accumulated evidences indicated that *TCP* genes were involved in a broad range of growth-related processes, such as flower and leaf shape, axillary meristem development, shoot branching, gametophyte development, hormone signaling, seed germination, regulation of the circadian clock, and defense (Kosugi and Ohashi, 1997, 2002; Martín-Trillo and Cubas, 2009; Danisman et al., 2012; Manassero et al., 2013; Lucero et al., 2017; Shang et al., 2020).

Given the critical roles in diverse biological processes, *TCP* genes have been identified in various plants, especially in angiosperms, which usually harbors more than 20 members (Liu et al., 2019). The evolutionary history of the *TCP* gene family (especially CYC clade) have been well characterized in angiosperms. Most previous studies have confirmed that the family can be classified into Class I (also known as PCF class or TCP-P class) and Class II (also known as TCP-C class) in angiosperms (Yao et al., 2007; Mondragón-Palomino and Trontin, 2011; Liu et al., 2019). Class I has a conserved four-amino-acid deletion in the TCP domain. Class II can be divided into the CYC clade and the CINCINNATA (CIN) clade (Martín-Trillo and Cubas, 2009; Liu et al., 2019). The CYC clade underwent two duplication events in the core eudicots, leading to three subgroups: CYC1, CYC2, and CYC3 (Howarth and Donoghue, 2006; Chapman et al., 2008). The CYC1 subclade includes maize TB1, which was related to the control of shoot branching (Doebley et al., 1997). CYC2, which includes *Anthirrinum CYC*, had a key role in the evolution of floral dorsoventral asymmetry (zygomorphy) (reviewed in Busch and Zachgo, 2009; Hileman, 2014; Fambrini and Pugliesi, 2017). It was reported that the CYC2 subclade has expanded by duplication events in many species-rich taxa with zygomorphic flowers such as Asterales, Dipsacales, Fabales, Lamiales, Proteales, and Ranales (Citerne et al., 2003, 2017; Gübitz et al., 2003; Reeves and Olmstead, 2003; Howarth and Donoghue, 2005; Kölsch and Gleissberg, 2006; Chapman et al., 2008; Howarth et al., 2011; Tähtiharju et al., 2012; Yang et al., 2012; Bello et al., 2017). The CYC3 subclade contains genes such as *Arabidopsis BRANCHED2* (*BRC2*), expressed both in branch and flower primordia, and also appears to be related with the control of shoot branching or the flower development (Martín-Trillo and Cubas, 2009).

Viridiplantae (green plants) contains green algae (Chlorophytes), streptophyte algae (Charophytes), and land plants (Embryophytes). The streptophyte algae is paraphyletic and comprises six lineages, i.e., Klebsormidiophyceae, Chlorokybophyceae, Mesostigmatophyceae, Zygnematophy- ceae, Coleochaetophyceae, and Charophyceae. Of them, Zygnematophyceae has been recognized as the most likely sister group of extant embryophytes (land plants) (Cheng et al., 2019; One Thousand Plant Transcriptomes Initiative, 2019). Land plant harbors five major lineages, i.e., bryophytes, lycophytes, ferns, gymnosperms, and angiosperms (One Thousand Plant Transcriptomes Initiative, 2019; Zhang et al., 2022). In comparison to the vast body of work developed in angiosperms, the evolutionary history of *TCP* genes in other lineages of green plants has not been widely investigated. Based on the BLAST searches against the EST database or PCR amplification in non-angiosperm plant species, Navaud et al. (2007) firstly reported that *TCP* genes may have originated before land plant emergence and form small families of no more than 10 members in pluricellular green algae (*Cosmarium* and *Chara*), bryophytes (*Physcomitrella*), ferns (*Ceratopteris* and *Equisetum*), lycophytes (*Selaginella*), and gymnosperms (*Pinus*, *Picea*, *Cycas*, and *Gnetum*). Liu et al. (2019) investigated the evolutionary history of the *TCP* gene family of land plant based on the genome-wide analysis and found that *TCP* genes might emerged at the ancestor of land plants and expanded significantly through whole-genome duplication (WGD). Although 47 species with whole genome sequence (including 25 algae and 22 land plants) were searched in their study, the representative species of streptophyte algae, especially the sister group of land plants (Zygnematophyceae), were overlooked. And also, of 22 land plant species, only 4 non-flowering plant species had been surveyed. Nevertheless, phylogenetic analysis in previous studies indicated that both Class I and CIN *TCP* genes were identified in all the investigated species, while the CYC clade seemed to be absent in non-flowering species (Navaud et al., 2007; Martín-Trillo and Cubas, 2009; Liu et al., 2019). In addition, the *TCP* gene family was likely to have undergone a large expansion over the course of evolution (Navaud et al., 2007; Liu et al., 2019). However, due to the sparse sampling or uncompleted genome data in these non-flowering species, when and how the *TCP* gene family expanded and diversified, especially the divergence of Class II and the origin of CYC, were still controversial.

The recent upward trend in the number of completely sequenced genomes or transcriptomes in different phylogenetic lineages give us the opportunity to revisit the evolutionary history of the *TCP* gene family and investigate how *TCP* genes expanded and diverged. In this study, an extensive survey of *TCP* genes was firstly conducted for some representative species with public genome data across all major lineages of green plants, especially the non-flowering plants. Considering the unevenly distribution of whole genome sequencing across clades, some transcriptomes were also selected for gymnosperms and ferns, which greatly expand coverage across the green plant clade and sampling density within many key clades. Then, a detailed phylogenetic analysis of all available sequence information was performed to infer their origin, diversification and expansion. Lastly, chromosomal locations, gene duplication, and synteny analysis were performed to investigate the expansion mechanism.

## Materials and methods

### Identification of *TCP* genes

**FIGURE 1:**
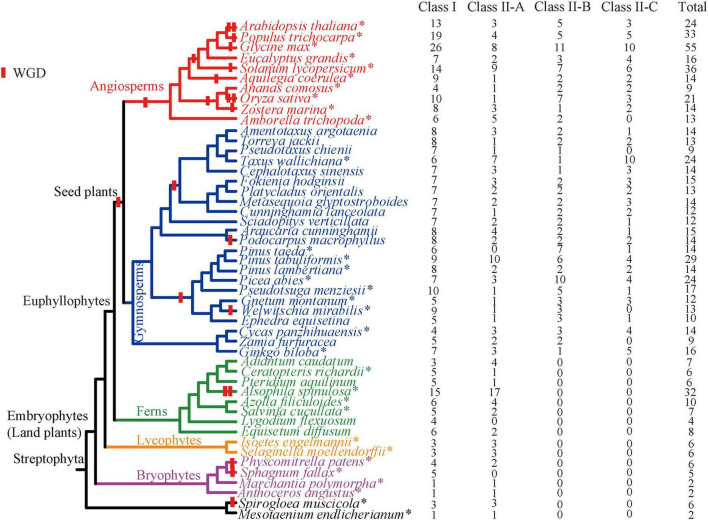
Copy number of the *TCP* gene family for all investigated species in this study. Species with whole genome sequences are represented by asterisks. The overall topology of these species is based on One Thousand Plant Transcriptomes Initiative (2019), decorated with whole-genome duplication events (Rensing et al., 2007, 2008; Jiao et al., 2011; Li et al., 2015; Ren et al., 2018; Cheng et al., 2019; Stull et al., 2021; Huang et al., 2022).

A total of 59 species, including red algae, green algae, streptophyte algae, bryophytes, lycophytes, ferns, gymnosperms, and angiosperms, were surveyed in our study (Figure 1 and Supplementary Table 1). Firstly, the coding and genomic sequences of *TCP* genes of 14 species were retrieved according to the gene ID (Supplementary Table 1 in Liu et al., 2019) from phytozome database,^1^ including 4 non-flowering plants (moss: *Physcomitrella patens*, *Sphagnum fallax*, *Marchantia polymorpha*; lycophyte: *Selaginella moellendorffii*), and 10 flowering species representing all major lineages of angiosperms according to APG IV (The Angiosperm Phylogeny Group, 2016). Then, the genomic sequences, coding sequences (CDS) and annotations of other 16 genomes of land plants (including 10 gymnosperms, 4 ferns, 1 lycophyte, and 1 bryophyte) were retrieved from phytozome, fernbase,^2^ CNGB,^3^ NGDC,^4^ NCBI,^5^ TreeGenes,^6^ Plantgenie,^7^ or figshare^8^ database (Supplementary Table 1). To increase the sampling density of gymnosperm and ferns representatives, 17 transcriptomes were also download from NCBI. Thirdly, the Hidden Markov model (HMM) profile of the conserved TCP domain (PF03634) was downloaded from the Pfam database^9^ and hmmsearch was performed using the hmmer 3.0^10^ with an *e*-value of 0.01. BLASTP against all protein sequences in each genome with the amino acid sequences of *Arabidopsis thaliana*, were also conducted with an *e*-value of 10. In order to infer the origin of the *TCP* gene family, attempts to identify *TCP* genes from green algae (*Botryococcus braunii*, *Chlamydomonas reinhardtii*, *Chromochloris zofingiensis*, *Coccomyxa subellipsoidea*, *Dunaliella salina*, *Micromonas pusilla* CCMP1545, *Micromonas* sp. RCC299, *Ostreococcus lucimarinus* and *Volvox carteri*), red algae (*Porphyra umbilicalis*) and two early diverging streptophyte algae (*Spirogloea muscicola* and *Mesotaenium endlicherianum*) were also made. Subsequently, all hits obtained from HMM and BLAST searches were merged together, and the redundant hits were removed. The annotation errors of some *TCP* genes were manually corrected based on the BLAST results. Finally, all candidate sequences were confirmed to be *TCP* genes by blast against the Pfam database.

### Sequence alignments and phylogenetic analysis

The coding regions of *TCP* genes were aligned using Clustal X (Thompson et al., 1997) and manually adjusted in Bioedit v7.0.9 (Hall, 1999). The alignment logos of the TCP domain were generated with SeqLogo in TBtools (Chen et al., 2020). Additionally, the construction of a reliable phylogenetic tree of TCP proteins is problematic due to the small size (59 amino acids maximum) of the conserved TCP domain sequence. Therefore, we aligned the maximum number of amino acids for each protein. To comprehensively explore the evolutionary history of the *TCP* gene family in green plants, both Maximum-Likelihood (ML) analysis and Bayesian Inference (BI) were performed based on the all conserved nucleotide and amino acid sequences. The ML tree was generated by IQ-TREE v1.6.8 (Nguyen et al., 2014), using the best-fit model selected by ModelFinder (Kalyaanamoorthy et al., 2017) and with 5,000 replications of ultrafast bootstrap support values (UFBoot) (Minh et al., 2013). The BI analysis was conducted with MRBAYES 3.2.6 (Ronquist et al., 2012) by running four simultaneous Markov chain Monte Carlo (MCMC) simulations, sampling every 100 generations, and discarding the first 2,500 trees as “burn-in.” The remaining trees were used to calculate posterior probabilities using a majority-rule consensus. Tracer v1.7 (Rambaut et al., 2018) was used to check for convergence and to ensure that effective sample sizes (ESS) were >200 for all parameters. The final trees were visualized or manually improved by the online program iTOL.^11^

### Chromosomal location, gene duplication, and synteny analysis

To infer the expansion mechanism of the *TCP* gene family, gene duplication and synteny analysis were performed for six species (ferns: *Alsophila spinulosa*; gymnosperms: *Ginkgo biloba*, *Cycas panzhihuaensis*, and *Pinus tabuliformis*; and angiosperms: *A. thaliana* and *O. sativa*) as follows. Firstly, GFF files, gene files, targeted *TCP* genes of these six species were downloaded or extracted. Secondly, BLASTP was performed to search homologous sequences in each species with *e* < 1e−5 and the top five self-BLASTP hits were considered as candidate duplicated genes. Then, the intra-genomic syntenic analysis were conducted using MCScanX (Wang et al., 2012) in TBtools with default parameters through the above BLASTP searches. The “*duplicate gene classifier*” program implemented in the MCScanX was employed to identify the replication mode. Genes can be classified into singletons, dispersed duplicates, proximal duplicates, tandem duplicates, and segmental duplicates/WGD depending on their copy number and genomic distribution (Wang et al., 2012). Lastly, the location of *TCP* genes and the duplicated genes caused by tandem and segmental duplications/WGD were displayed in TBtools.

## Results

### Phylogenetic analyses

The copy number of the *TCP* gene family is greatly variable in green plants (Figure 1). No homologs were identified in nine green algae and one red algae. Among the other 49 species with *TCP* genes, a total of 691 *TCP* genes were obtained (Figure 1 and Supplementary Table 1). Of them, 437 TCPs were newly identified. The two early diverging algae, *S. muscicola* and *M. endlicherianum*, possess only two and six members, respectively. The bryophytes, early diverging clade of land plants (including hornworts, liverworts, and mosses) harbors two to six members. The lycophytes *S. moellendorffii* and *Isoetes engelmannii* possess six members. In ferns, most species harbor no more than 10 members, with the exception of *A. spinulosa*, in which 32 members were identified. It should be noted that the copy number of *TCP* genes in ferns were likely to be underestimated, because only transcriptomes were searched for *Adiantum caudatum*, *Equisetum diffusum*, *Lygodium flexuosum*, and *Pteridium aquilinum*. There was an obvious expansion for the *TCP* gene family in seed plants (Figure 1). The gymnosperms possess an average of over 15 members, with *Zamia furfuracea*, *Pseudotaxus chienii* having the least (9 genes) and *P. tabuliformis* having the most (29 genes). The copy number of the *TCP* gene family of angiosperms vary greatly, ranging from 9 (*Ananas comosus*) to 55 (*Glycine max*), with an average of 23.5 members (Figure 1). In general, a gradually expansion history of the *TCP* gene family was observed during plant evolution, as the gene number continued to increase from algae to angiosperms.

**FIGURE 2:**
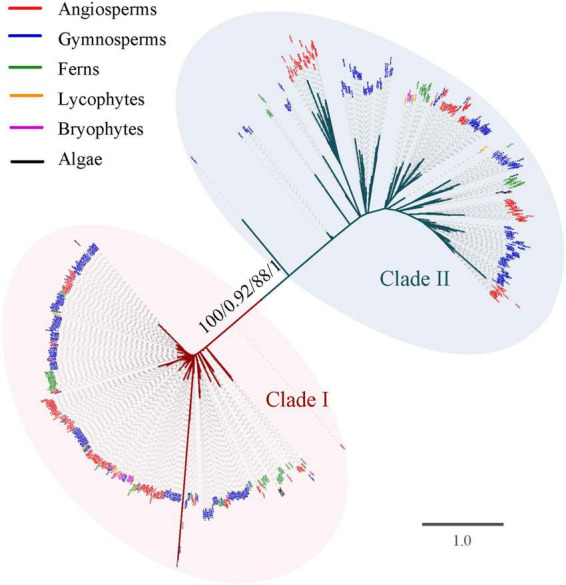
Phylogenetic tree of the *TCP* gene family constructed based on the amino acid sequences of the TCP domain. The tree was not rooted. Branch lengths indicate the number of amino acid residues substitutions per site and are drawn to scale. Numbers associated with nodes are UFBoot support values and posterior probability in the phylogeny analysis based on amino acid sequences (first and second) and CDS (third and fourth), respectively. All species abbreviations are listed in Supplementary Table 1.

**FIGURE 3:**
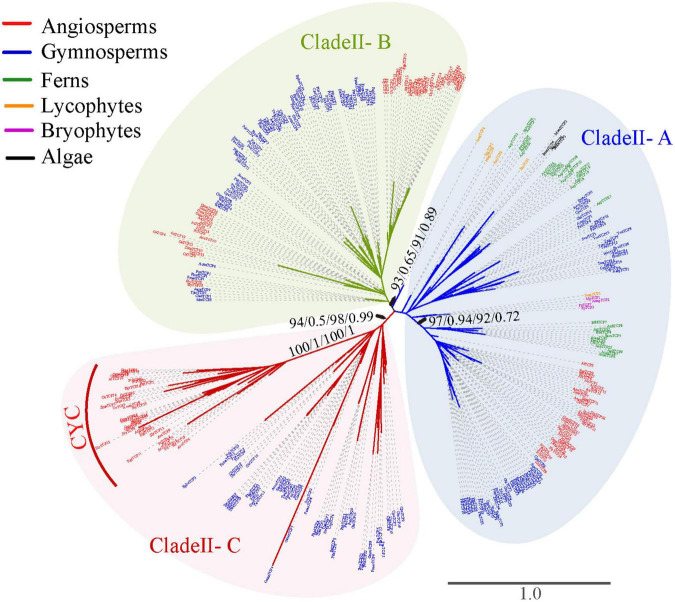
Phylogenetic tree of Class II *TCP* genes constructed based on the amino acid sequences of the TCP domain and the upstream conserved sequences. The tree was not rooted. Branch lengths indicate the number of amino acid residues substitutions per site and are drawn to scale. Numbers associated with nodes are UFBoot support values and posterior probability in the phylogeny analysis based on amino acid sequences (first and second) and CDS (third and fourth), respectively. All species abbreviations are listed in Supplementary Table 1.

To explore the evolutionary history of the *TCP* gene family, phylogenetic analysis was firstly performed based on the alignment nucleotide and amino acid sequence of the TCP domain, respectively. Both the unrooted phylogenetic trees inferred from nucleotide and amino acid sequences with ML and BI analysis confirmed the classification of Class I and Class II (Figure 2). The Class I was slightly larger than Class II (348 vs. 343 members). All the investigated *Streptophyta* species (including land plants and two Zygnematophyceae) had *TCP* genes from both classes, except for *S. fallax* and *L. flexuosum*, which had no Class II *TCP* genes (Figure 1 and Supplementary Table 1). Class II was mainly divided into three subclades supported by moderate or high bootstrap values (A–C), although their relationship was uncertain (Figure 3). Class II-A contained gene members belonging to all phylogenetic groups from moss to angiosperms, while Class II-B and Class II-C only harbored sequences from seed plants (gymnosperms and angiosperms) (Figures 1, 3). In Class II-A, one subgroup, which harbors the sequence from angiosperms, gymnosperms and ferns, was highly supported (support value = 97/0.94/92/0.72) (Figure 3). In Class II-C, one highly supported subgroup (CYC) was recognized (support value = 100/1/100/1) (Figure 3). It should be noted that all seed plants possess three subclades of Class II, except for *Amborella trichopoda*, *Pinus taeda*, *P. chienii*, *Welwitschia mirabilis*, and *Z. furfuracea*, in which Class II-A or Class II-C was absent (Supplementary Table 1). Although the phylogenetic position of *TCP* genes in lycophytes, and algae were largely unresolved due to the limited informative sites of the conserved domain, the subdivision of *TCP* genes in euphyllophytes was further supported by the phylogenetic analysis for angiosperms, gymnosperms and ferns, respectively (Supplementary Figures 1–3).

### Sequence characteristics of TCP domain

**FIGURE 4:**
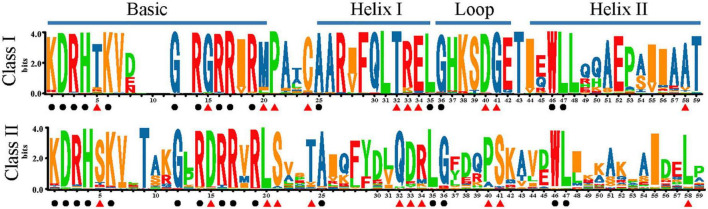
Sequence logos of the plant TCP domains. Bit scores represents the relative frequency of amino acids. The black circle represents conserved loci within the whole family. The red triangle indicates divergent but conserved residues in each subclade.

Using SeqLogo, we obtained a graphical representation of sequence variation of TCPs, which provided a more precise description of polymorphism sites and conserved sites (Figure 4). The relative frequency of corresponding amino acids is reflected by the height of the symbol at each position. The TCP domain commonly consisted of a basic region and a helix-loop-helix (HLH) structure. The basic region of the TCP domain, was highly conserved in all family members, with two consensus sequences, DRHxK and RxRRxR (Figure 4). For the HLH region, only five sites were highly conserved, including Ala (A)-25, Leu (L)-35, Gly (G)-36, Trp (W)-46, and L-47 (Figure 4). The most striking difference between these two classes is a four-amino acid deletion in the basic region of Class I relative to Class II. In addition, Class I and Class II contain distinct residues at positions 5, 15, 20, 21, 24, 32, 33, 34, 40, 41, and 58 (Figure 4). In general, the residues in Class I were relatively more conserved than Class II. For Class II, the most striking difference among three subgroups (A, B, and C) was located in the upstream of TCP domain (Supplementary Figure 4). And the CYC subclade and other subclade (Class II-A, Class II-B, and gymnosperms of Class II-C) possessed distinct residues at positions 16, 33, 35, 48, and 49 (Supplementary Figure 4).

### Chromosomal location, gene duplication, and synteny analysis of *TCP* genes

**FIGURE 5:**
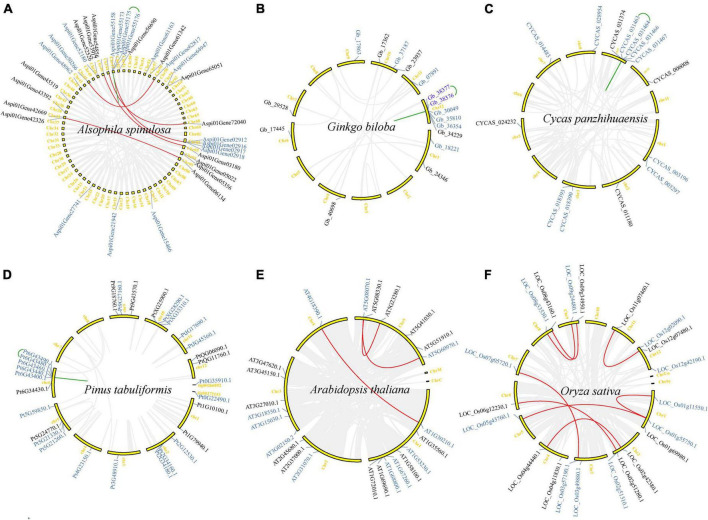
Synteny analysis of *TCP* genes in six representative species **(A–F)**. Gene names on the side of each chromosome correspond to the approximate locations of each *TCP* gene. The detailed information is listed in Supplementary Table 1. Gray lines indicate all synteny blocks in the whole genome of each species. Red and green lines suggest *TCP* gene pairs with segmental/WGD duplication and tandem duplication, respectively. Gene names with black and blue mean Class I and Class II *TCP* genes, respectively.

All identified *TCP* genes in *A. spinulosa*, *G. biloba*, *C. panzhihuaensis*, *P. tabuliformis*, *A. thaliana*, and *O. sativa* were mapped to the chromosomes or some scaffolds (Figure 5). *TCP* genes are widely distributed throughout genomes but are uneven among chromosomes. In *A. thaliana*, all five chromosomes harbor *TCP* genes, but eight of the 24 *TCP* genes are located on Chr (chromosome) 1 and only one gene was detected in Chr 4 (Figure 5E). In other species, *TCP* genes are not found on all chromosomes. For instance, 32 *TCP* genes of *A. spinulosa* were located in 22 chromosomes (a total of 69 chromosomes) (Figure 5A). Chr 2, 3, 5, and 8 of *G. biloba* (Figure 5B), Chr 4, 6, and 11 of *C. panzhihuaensis* (Figure 5C), Chr 7 and 8 of *P. tabuliformis* (Figure 5D), and Chr 10 of *O. sativa* (Figure 5F) do not carry any *TCP* genes.

Gene duplication and synteny analysis indicated that only a few of *TCP* genes in *A. thaliana* (4), *G. biloba* (2) and *C. panzhihuaensis* (1) were identified as singleton (Table 1). Most *TCP* genes (129/136) were generated by gene duplication events. More than half (73/136) of *TCP* genes were recognized as dispersed duplications and nearly one fourth (32/136) were recognized as segmental duplications/WGD. In *A. spinulosa*, all four types of gene duplications were detected, and dispersed and segmental duplications/WGD were the two main types, with 16 (50%) and 11 (34%), respectively (Table 1). In *G. biloba*, 10 (63%), 2 (13%), and 2 (13%) *TCP* genes are generated by dispersed, proximal, and segmental duplications/WGD, respectively. In *C. panzhihuaensis*, 9 (64%), 2 (14%), and 2 (14%) *TCP* genes are generated by dispersed, proximal, and segmental duplications/WGD, respectively. In *P. tabuliformis*, 18 (62%), 9 (31%), and 2 (7%) *TCP* genes are generated by dispersed, proximal, and tandem duplications, respectively. In *A. thaliana*, 14 (58%) and 6 (25%) *TCP* genes are generated by dispersed and segmental duplications/WGD, respectively. In *O. sativa*, 6 (29%) and 15 (71%) *TCP* genes are generated by dispersed and segmental duplications/WGD, respectively. Our results indicated that both dispersed duplications and segmental duplications/WGD play a critical role in the expansion of the *TCP* gene family.

**TABLE 1:** Numbers of *TCP* genes from different origins as classified by *duplicate gene classifier* in six representative euphyllophytes genomes.

Group	Species	Total	Singleton	Dispersed	Proximal	Tandem	WGD or segmental
Ferns	*Alsophila spinulosa*	32	0	16 (9/7)	3 (0/3)	2 (0/2)	11 (6/5)
Gymnosperms	*Cycas panzhihuaensis*	14	1 (0/1)	9 (3/6)	2 (1/1)	2 (0/2)	0
	*Ginkgo biloba*	16	2 (1/1)	10 (6/4)	2 (0/2)	2 (0/2)	0
	*Pinus tabuliformis*	29	0	18 (9/9)	9 (0/9)	2(0/2)	0
Angiosperms	*Arabidopsis thaliana*	24	4 (1/3)	14 (10/4)	0	0	6 (2/4)
	*Oryza sativa*	21	0	6 (2/4)	0	0	15 (8/7)

The numbers in the brackets indicate Class I and Class II TCP genes, respectively.

## Discussion

### The origin and diversification of the *TCP* gene family

In this study, we present a genome-wide survey of *TCP* genes in 59 species (42 genomes and 17 transcriptomes), ranging from algae to angiosperms, and reconstruct the evolutionary history of this gene family. It was reported that *TCP* genes were present in *Chara* (Charophyceae) and *Cosmarium* (Zygnematophyceae) (Navaud et al., 2007). In our study, *TCP* genes were newly identified in other two streptophyte algae (*S. muscicola* and *M. endlicherianum*), but no homologs were identified in any species of Chlorophyta and Rhodophyta. Additionally, we also tried to identify *TCP* genes in the genomes or transcriptomes of other streptophyte algae by TBLASTN in NCBI, and no sequences were identified. All these evidences confirmed that the origin of the *TCP* gene family predates the emergence of land plants, possibly in the common ancestor of Phragmoplastophyta (Zygnematophyceae, Coleochaetophyceae, Charophyceae, and all extant land plants) (Navaud et al., 2007). However, Classes I and II genes were clearly diverged in all these species, even in the early divergent algal species. Not any possible common ancestor of *TCP* genes was identified in this study, although extensive searches within prokaryotic and eukaryotic genomes were performed, which renders it difficult to speculate from which *TCP* genes arose. We tentatively hypothesized that two types of *TCP* genes were likely generated simultaneously, and then evolved independently. Nevertheless, because of the sparse sampling in the basic group of green plants, an alternative hypothesis could not be completely ruled out. The scenario is that the ancestral *TCP* gene might have been present in lineages that have since disappeared. For these reasons, we suggest that increasing sampling in early divergent streptophyte algae (such as Zygnematophyceae and Charophyceae) is a requirement for further confirmation of the origin of the *TCP* genes in the future.

The *TCP* gene family was generally divided into Classes I and II, with Class II being further divided into CIN and CYC clades in angiosperms (Navaud et al., 2007; Martín-Trillo and Cubas, 2009; Liu et al., 2019). Consistent with most previous studies, the topology of the phylogenetic tree of the 691 *TCP* genes was also preliminary divided into two Class I and Class II with relatively high support values (Figure 2). Unlike with previous studies, our phylogenetic tree newly recognized three subclades (A, B, and C) in Class II (Figure 3), possibly resulted from the increasing sampling of non-flowering plants. The previously described CYC subclade was embedded in Class II-C, and CIN subclade was assigned into Class II-A and Class II-B subclades (Figure 3). The non-spermatophyte (algae, bryophytes, lycophytes, and ferns) only harbor Class II-A, and seed plants (gymnosperms and angiosperms) possess all three types (Figure 3). Although the relationship of three subclades of Class II was not resolved, the Class II-A subclade was likely more related to the ancestral Class II *TCP* genes, and two ancient gene duplications predating the divergence of seed plants possibly gave rise to Class II-B and Class II-C. The origin of *CYC* was most likely coincided with the occurrence and diversification of flowers in angiosperms and the subsequent gene loss likely resulted in the absent of *CYC* genes in the basal angiosperms *A. trichopoda* (Figure 2 and Supplementary Table 1). However, we could not totally rule out the possibility that the origin of *CYC* would take place after the split of *Amborella* with other angiosperms. The ancient gene duplications of Class II and the origin of *CYC* were likely related with the ancestral WGD in the common ancestor of extant seed plants (Jiao et al., 2011). Class II *TCP* genes had crucial roles in the development of reproductive organs (such as gametophyte and flower) (Pagnussat et al., 2005; Martín-Trillo and Cubas, 2009). The retention of three subclades of Class II in the ancestor of seed plants might be related with the origin of seed and flower, ultimately, promoting the origin and rapid diversification of the angiosperms.

Additionally, given the results of sequence comparison (Figure 4) and phylogenetic analysis (Figures 2, 3 and Supplementary Figures 1–3), Class I *TCP* genes were relatively more conserved than Class II, which suggested that Class I was probably under strong purifying selection and Class II might had gone through prominent functional differentiation over the course of evolution. It has been reported that Class I promote the growth and proliferation in angiosperms. By contrast, the role of Class II was much more complicated and diverged, which was reported to participate in the development of leaf, flower, shoot branching, or even hormone signaling (Doebley et al., 1997; Kosugi and Ohashi, 1997, 2002; Busch and Zachgo, 2009; Martín-Trillo and Cubas, 2009; Danisman et al., 2012; Hileman, 2014; Fambrini and Pugliesi, 2017). In the bryophyte *P. patens*, *PpTCP5* (Class II) was reported to be involved in regulating sporophyte branching (Ortiz-Ramírez et al., 2016), similar to the well-known function of members of the Class II *TCP* genes of maize in shoot branching (Doebley et al., 1997), although their branching structures (axillary meristems or branch initials) are very different. A wider and more specific expression profile of *TCP* genes in seed plants indicated their functional divergence. In the bryophytes (*M. polymorpha* and *P. patens*), both Class I and Class II had high expression levels in many tissues or organs across various development stages (Supplementary Figures 3A,B of Liu et al., 2019). In seed plants, the vast majority of Class I and Class II-A *TCP* genes had a broader expression profile (Supplementary Figure 5). However, more members of Class II-B and Class II-C *TCP* genes tend to had limited expression, such as *AthTCP1*, *AthTCP12*, *AthTCP18* of *Arabidopsis* and *PabTCP5*, *PabTCP20*, *PabTCP23*, *PabTCP24* of *Picea* (Supplementary Figure 5), which indicated that they might perform specific roles. As plant complexity increased in the ancestor of seed plants, more intricate regulatory networks were needed. Our study revealed that the expansion and diversification of *TCP* genes (especially Class II) in green plants might be possibly associated with the development of new organs (e.g., seed, leaf, branching, and flower) or the increasing organ complexity (e.g., floral dorsoventral asymmetry) (Floyd and Bowman, 2007). Additionally, the lineages or species-specific clade was frequently observed in gymnosperms and angiosperms (Figures 2, 3 and Supplementary Figures 1, 2), indicating that *TCP* genes might have experienced distinct evolution history in each lineage, which was possibly related with the adaptation to the distinct environment. Over the course of evolution, frequent gene and genome duplication events and subsequent functional divergence have significantly contributed to the diversification of *TCP* genes, which could further facilitate plant diversification, adaptation, and evolution.

### The expansion of the *TCP* gene family

Gene duplication is a major driving force in the expansions and diversification of TF family, which might be related with the evolution of the innovative traits (Taylor and Raes, 2004; Floyd and Bowman, 2007). After gene duplication, plants take up removing most redundant gene copies in a long evolutionary process, but some duplicated copies are retained depending on their environmental adaptation, which is the cause of copy number variation among different species. According to our comprehensive survey, both Classes I and II *TCP* genes experienced a continuous expansion and grew from a few members in algae, moss and lycophytes to dozens, and sometimes over 50 members in angiosperms (Figure 1). In addition, our comprehensive study from a wide phylogenetic scale and intensive phylogenetic analysis indicated that at least four subclades (Class I and three subclades of Class II) have been occurred in the ancestor of spermatophyte (Figure 3). All these evidences indicated that the *TCP* gene family gradually expanded and diversified during the diversification of green plants, especially in streptophytes.

Gene duplication and synteny analysis indicated that most *TCP* genes (129/136) were generated by gene duplication events. In different groups, the expansion of the *TCP* gene family might be caused by different ways. The relative larger members of the *TCP* gene family in *S. muscicola* (algae) and *P. patens* (moss) than other species in the same lineages (Figure 1) might be result from the recent WGD (Rensing et al., 2007, 2008; Cheng et al., 2019), which confirmed by the monophyly of Classes I and II genes in each species (Figures 2, 3). *A. spinulosa*, which could have undergone two WGD events (Huang et al., 2022), also harbors the most striking large number of *TCP* genes in ferns (Figure 3). Our results indicated that both dispersed duplications and segmental duplications/WGD play a critical role in expansion of the *TCP* gene family in *A. spinulosa* (Table 1). In gymnosperms, although an ancient WGD has been reported in the common ancestor of extant gymnosperms, few recent WGD events were detected (Li et al., 2015; Guan et al., 2016; Liu et al., 2022; Niu et al., 2022). This was likely associated with the lacking of segmental duplications/WGD in *G. biloba*, *C. panzhihuaensis*, and *P. tabuliformis*, and more than 60% of the gymnosperms *TCP* genes were generated by the dispersed duplication (Table 1). By contrast, both ancient and recent WGD were occurred in most extant angiosperms (Jiao and Paterson, 2014; Ren et al., 2018), which result in the expansion of the *TCP* gene family in angiosperms, and this view was also supported by previous study (Liu et al., 2019). It is noteworthy that in some species (e.g., *A. trichopoda* and *A. comosus*), the copy number was significantly less than other species of angiosperms, which could be associated with the lacking of recent WGD in these species (Amborella Genome Project, 2013; Ming et al., 2015). Except for segmental duplications/WGD, our study also detected a high dispersed duplication rate in *A. thaliana* and *O. sativa* (Table 1). Therefore, it can be concluded that the expansion of the *TCP* gene family in green plants was mainly driven by dispersed duplication, segmental duplications/WGD.

## Data availability statement

The datasets presented in this study can be found in online repositories. The names of the repository/repositories and accession number(s) can be found in the article/Supplementary material.

## Author contributions

Y-YL and S-LK planned and designed this study. J-LW, Y-YL, H-WW, and Y-NC collected and analyzed the data. Y-YL, J-LW, S-LK, and H-WW wrote the manuscript. All authors contributed to the article and agreed to submit this version of the manuscript.

## Footnotes

1
http://phytozome.jgi.doe.gov/

2
https://www.fernbase.org/

3
https://db.cngb.org/

4
https://ngdc.cncb.ac.cn/

5
https://www.ncbi.nlm.nih.gov/

6
https://treegenesdb.org/

7
https://plantgenie.org/

8
https://figshare.com

9
http://pfam.xfam.org/

10
http://hmmer.org

11
https://itol.embl.de/
