# Supplementary material for: Comprehensive evolutionary analysis of the TCP gene family: Further insights for its origin, expansion, and diversification
Source: Front Plant Sci. 2022 Sep 2;13:994567. doi: 10.3389/fpls.2022.994567 (PMC9480096; doi:10.3389/fpls.2022.994567)
Supplement: Supplementary file 1 [file Data_Sheet_1.pdf]

Supplementary Material

Supplementary Figures

Supplementary Figure 1. Phylogenetic tree of *TCP* genes in angiosperms constructed based on the amino acid sequences of the TCP domain and its upstream and downstream conserved regions. The tree was not rooted. Branch lengths indicate the number of amino acid residues substitutions per site and are drawn to scale. Numbers associated with nodes are UFBoot support values and posterior probability in the phylogeny analysis based on amino acid sequences (first and second) and coding sequences (CDS) (third and fourth) of the domain, respectively. All species abbreviations are listed in Supplementary Table 1.

Supplementary Figure 2. Phylogenetic tree of *TCP* genes in gymnosperms constructed based on the amino acid sequences of the TCP domain and its upstream and downstream conserved regions. The tree was not rooted. Branch lengths indicate the number of amino acid residues substitutions per site and are drawn to scale. Numbers associated with nodes are UFBoot support values and posterior probability in the phylogeny analysis based on amino acid sequences (first and second) and CDS (third and fourth) of the domain, respectively. All species abbreviations are listed in Supplementary Table 1.

Supplementary Figure 3. Phylogenetic tree of *TCP* genes in ferns constructed based on the amino acid sequences of the TCP domain and its upstream and downstream conserved regions. The tree was not rooted. Branch lengths indicate the number of amino acid residues substitutions per site and are drawn to scale. Numbers associated with nodes are UFBoot support values and posterior probability in the phylogeny analysis based on amino acid sequences (first and second) and CDS (third and fourth) of the domain, respectively. All species abbreviations are listed in Supplementary Table 1.

Supplementary Figure 4. Sequence logos of the conserved sequences of Class II *TCP* genes. Bit scores represents the relative frequency of amino acids. The black circle represents conserved loci within the whole family. The red triangle indicates the distinct residues between CYC subclade and other three subclades.

Supplementary Figure 5. Expression profiles of *TCP* genes in *Arabidopsis thaliana* (A) and *Picea abies* (B). The expression data was retrieved from Arabidopsis RNA-seq database (http://ipf.sustech.edu.cn/pub/athrna/) and Plantgenie database (https://plantgenie.org/). No

expression data was detected for *PabTCP4* (Class II-B), *PabTCP6* (Class II-A) and *PabTCP10* (Class II-A).

Supplementary Tables

Supplementary Table 1. The detailed information of candidate *TCP* genes identified in this study.

Group	Species	Code	Classification	Gene	Duplication	Database* or reference
**Angiosperms**	*Arabidopsis thaliana*	AtTCP1/CYC/TB1	Class II-C	At1g67260	Dispersed	Liu et al., 2019
		AtTCP2	Class II-B	At4g18390	WGD or Segmental	Liu et al., 2019
		AtTCP3	Class II-A	At1g53230	Dispersed	Liu et al., 2019
		AtTCP4/MEE35	Class II-A	At3g15030	Singleton	Liu et al., 2019
		AtTCP5	Class II-B	At5g60970	WGD or Segmental	Liu et al., 2019
		AtTCP6	Class I	At5g41030	Dispersed	Liu et al., 2019
		AtTCP7	Class I	At5g23280	WGD or Segmental	Liu et al., 2019
		AtTCP8	Class I	At1g58100	Dispersed	Liu et al., 2019
		AtTCP9	Class I	At2g45680	Dispersed	Liu et al., 2019
		AtTCP10	Class II-A	At2g31070	Dispersed	Liu et al., 2019
		AtTCP11	Class I	At2g37000	Dispersed	Liu et al., 2019
		AtTCP12/BRC2	Class II-C	At1g68800	Singleton	Liu et al., 2019
		AtTCP13/PTF1	Class II-B	At3g02150	Dispersed	Liu et al., 2019
		AtTCP14	Class I	At3g47620	Dispersed	Liu et al., 2019
		AtTCP15	Class I	At1g69690	Dispersed	Liu et al., 2019
		AtTCP16	Class I	At3g45150	Dispersed	Liu et al., 2019
		AtTCP17	Class II-B	At5g08070	WGD or Segmental	Liu et al., 2019
		AtTCP18/BRC1	Class II-C	At3g18550	Singleton	Liu et al., 2019
		AtTCP19	Class I	At5g51910	Dispersed	Liu et al., 2019
		AtTCP20	Class I	At3g27010	Singleton	Liu et al., 2019
		AtTCP21/CHE	Class I	At5g08330	WGD or Segmental	Liu et al., 2019
		AtTCP22	Class I	At1g72010	Dispersed	Liu et al., 2019
		AtTCP23	Class I	At1g35560	Dispersed	Liu et al., 2019
		AtTCP24	Class II-B	At1g30210	WGD or Segmental	Liu et al., 2019
	Amborella trichopoda	AtrTCP1	Class II-A	evm_27.model.AmTr_v1.0_scaffold000		Liu et al., 2019
		AtrTCP2	Class I	evm_27.model.AmTr_v1.0_scaffold000		Liu et al., 2019
		AtrTCP3	Class I	evm_27.model.AmTr_v1.0_scaffold000		Liu et al., 2019
		AtrTCP4	Class I	evm_27.model.AmTr_v1.0_scaffold000		Liu et al., 2019
		AtrTCP5	Class I	evm_27.model.AmTr_v1.0_scaffold000		Liu et al., 2019
		AtrTCP6	Class II-A	evm_27.model.AmTr_v1.0_scaffold000		Liu et al., 2019
		AtrTCP7	Class I	evm_27.model.AmTr_v1.0_scaffold000		Liu et al., 2019
		AtrTCP8	Class II-A	evm_27.model.AmTr_v1.0_scaffold000		Liu et al., 2019
		AtrTCP9	Class I	evm_27.model.AmTr_v1.0_scaffold000		Liu et al., 2019

	AtrTCP10	Class II-B	evm_27.model.AmTr_v1.0_scaffold000	Liu et al., 2019
	AtrTCP11	Class II-A	evm_27.model.AmTr_v1.0_scaffold001	Liu et al., 2019
	AtrTCP12	Class II-A	evm_27.model.AmTr_v1.0_scaffold001	Liu et al., 2019
	AtrTCP13	Class II-B	evm_27.model.AmTr_v1.0_scaffold001	Liu et al., 2019
Ananas	AcomTCP1	Class II-B	Aco002292.1	Liu et al., 2019
comosus	AcomTCP2	Class II-C	Aco003020.1	Liu et al., 2019
	AcomTCP3	Class I	Aco006659.1	Liu et al., 2019
	AcomTCP4	Class I	Aco010326.1	Liu et al., 2019
	AcomTCP5	Class II-A	Aco010666.1	Liu et al., 2019
	AcomTCP6	Class II-B	Aco012417.1	Liu et al., 2019
	AcomTCP7	Class I	Aco015741.1	Liu et al., 2019
	AcomTCP8	Class I	Aco021664.1	Liu et al., 2019
	AcomTCP9	Class II-C	Aco024489.1	Liu et al., 2019
Aquilegia	AcoTCP1	Class I	Aqcoe1G137200.1	Liu et al., 2019
coerulea	AcoTCP2	Class II-A	Aqcoe1G240000.1	Liu et al., 2019
	AcoTCP3	Class I	Aqcoe3G008000.1	Liu et al., 2019
	AcoTCP4	Class II-C	Aqcoe3G048600.1	Liu et al., 2019
	AcoTCP5	Class I	Aqcoe3G081500.1	Liu et al., 2019
	AcoTCP6	Class I	Aqcoe3G164300.1	Liu et al., 2019
	AcoTCP7	Class I	Aqcoe3G335300.1	Liu et al., 2019
	AcoTCP8	Class II-B	Aqcoe3G370100.1	Liu et al., 2019
	AcoTCP9	Class II-C	Aqcoe3G395500.1	Liu et al., 2019
	AcoTCP10	Class II-B	Aqcoe4G007100.1	Liu et al., 2019
	AcoTCP11	Class I	Aqcoe5G347900.1	Liu et al., 2019
	AcoTCP12	Class I	Aqcoe5G422600.1	Liu et al., 2019
	AcoTCP13	Class I	Aqcoe6G015800.1	Liu et al., 2019
	AcoTCP14	Class I	Aqcoe7G051800.1	Liu et al., 2019
Eucalyptus	EgrTCP1	Class I	Eucgr.A01143.1	Liu et al., 2019
grandis	EgrTCP2	Class I	Eucgr.A02843.1	Liu et al., 2019
	EgrTCP3	Class I	Eucgr.B00471.1	Liu et al., 2019
	EgrTCP4	Class II-B	Eucgr.B00608.1	Liu et al., 2019
	EgrTCP5	Class II-C	Eucgr.B00699.1	Liu et al., 2019
	EgrTCP6	Class I	Eucgr.B03427.1	Liu et al., 2019
	EgrTCP7	Class I	Eucgr.B03529.1	Liu et al., 2019
	EgrTCP8	Class I	Eucgr.D02422.1	Liu et al., 2019
	EgrTCP9	Class II-A	Eucgr.F01204.1	Liu et al., 2019
	EgrTCP10	Class I	Eucgr.F02587.1	Liu et al., 2019
	EgrTCP11	Class II-C	Eucgr.G02354.1	Liu et al., 2019
	EgrTCP12	Class II-B	Eucgr.I02038.1	Liu et al., 2019
	EgrTCP13	Class II-C	Eucgr.J01466.1	Liu et al., 2019
	EgrTCP14	Class II-A	Eucgr.K01089.1	Liu et al., 2019
	EgrTCP15	Class II-B	Eucgr.K02535.1	Liu et al., 2019
	EgrTCP16	Class II-C	Eucgr.K02654.1	Liu et al., 2019
Glycine	GmaTCP1	Class I	Glyma.01G045500.1	Liu et al., 2019
max	GmaTCP2	Class I	Glyma.02G105900.1	Liu et al., 2019
	GmaTCP3	Class I	Glyma.03G018800.1	Liu et al., 2019
	GmaTCP4	Class II-C	Glyma.04G152400.1	Liu et al., 2019

GmaTCP5	Class II-B	Glyma.04G161400.1	Liu et al., 2019
GmaTCP6	Class I	Glyma.04G170600.1	Liu et al., 2019
GmaTCP7	Class II-C	Glyma.05G013300.1	Liu et al., 2019
GmaTCP8	Class II-B	Glyma.05G019900.1	Liu et al., 2019
GmaTCP9	Class I	Glyma.05G027400.1	Liu et al., 2019
GmaTCP10	Class I	Glyma.05G050400.1	Liu et al., 2019
GmaTCP11	Class II-B	Glyma.05G142000.1	Liu et al., 2019
GmaTCP12	Class I	Glyma.06G193000.1	Liu et al., 2019
GmaTCP13	Class II-B	Glyma.06G204300.1	Liu et al., 2019
GmaTCP14	Class II-C	Glyma.06G210600.1	Liu et al., 2019
GmaTCP15	Class II-A	Glyma.06G232300.1	Liu et al., 2019
GmaTCP16	Class II-A	Glyma.06G284500.1	Liu et al., 2019
GmaTCP17	Class I	Glyma.07G080300.1	Liu et al., 2019
GmaTCP18	Class II-B	Glyma.08G097900.1	Liu et al., 2019
GmaTCP19	Class II-B	Glyma.08G247300.1	Liu et al., 2019
GmaTCP20	Class II-C	Glyma.08G256400.1	Liu et al., 2019
GmaTCP21	Class I	Glyma.08G299400.1	Liu et al., 2019
GmaTCP22	Class I	Glyma.09G284300.1	Liu et al., 2019
GmaTCP23	Class I	Glyma.09G284500.1	Liu et al., 2019
GmaTCP24	Class I	Glyma.10G057400.1	Liu et al., 2019
GmaTCP25	Class I	Glyma.10G240200.1	Liu et al., 2019
GmaTCP26	Class II-C	Glyma.10G246200.1	Liu et al., 2019
GmaTCP27	Class I	Glyma.10G285900.1	Liu et al., 2019
GmaTCP28	Class I	Glyma.11G196000.1	Liu et al., 2019
GmaTCP29	Class II-A	Glyma.12G121500.1	Liu et al., 2019
GmaTCP30	Class II-A	Glyma.12G158900.1	Liu et al., 2019
GmaTCP31	Class I	Glyma.12G168300.1	Liu et al., 2019
GmaTCP32	Class II-A	Glyma.12G208800.1	Liu et al., 2019
GmaTCP33	Class II-A	Glyma.12G228300.1	Liu et al., 2019
GmaTCP34	Class II-C	Glyma.13G047400.1	Liu et al., 2019
GmaTCP35	Class I	Glyma.13G144100.1	Liu et al., 2019
GmaTCP36	Class II-B	Glyma.13G219900.1	Liu et al., 2019
GmaTCP37	Class II-A	Glyma.13G271700.1	Liu et al., 2019
GmaTCP38	Class II-A	Glyma.13G292500.1	Liu et al., 2019
GmaTCP39	Class II-B	Glyma.15G092500.1	Liu et al., 2019
GmaTCP40	Class I	Glyma.16G004300.1	Liu et al., 2019
GmaTCP41	Class I	Glyma.16G053900.1	Liu et al., 2019
GmaTCP42	Class II-B	Glyma.17G079900.1	Liu et al., 2019
GmaTCP43	Class I	Glyma.17G099100.1	Liu et al., 2019
GmaTCP44	Class II-C	Glyma.17G121500.1	Liu et al., 2019
GmaTCP45	Class I	Glyma.17G132400.1	Liu et al., 2019
GmaTCP46	Class I	Glyma.18G121400.1	Liu et al., 2019
GmaTCP47	Class II-B	Glyma.18G268700.1	Liu et al., 2019
GmaTCP48	Class II-C	Glyma.18G280700.1	Liu et al., 2019
GmaTCP49	Class II-B	Glyma.19G030900.1	Liu et al., 2019
GmaTCP50	Class II-C	Glyma.19G044400.1	Liu et al., 2019

	GmaTCP51	Class I	Glyma.19G095300.1		Liu et al., 2019
	GmaTCP52	Class I	Glyma.20G001600.1		Liu et al., 2019
	GmaTCP53	Class I	Glyma.20G103400.1		Liu et al., 2019
	GmaTCP54	Class II-C	Glyma.20G148500.1		Liu et al., 2019
	GmaTCP55	Class I	Glyma.20G154400.1		Liu et al., 2019
Oryza	OsTCP1	Class II-A	LOC_Os01g11550.1	WGD or Segmental	Liu et al., 2019
sativa	OsTCP2	Class II-B	LOC_Os01g55750.1	WGD or Segmental	Liu et al., 2019
	OsTCP3	Class I	LOC_Os01g69980.1	Dispersed	Liu et al., 2019
	OsTCP4	Class I	LOC_Os02g42380.1	WGD or Segmental	Liu et al., 2019
	OsTCP5	Class I	LOC_Os02g51280.1	WGD or Segmental	Liu et al., 2019
	OsTCP6	Class II-B	LOC_Os02g51310.1	Dispersed	Liu et al., 2019
	OsTCP7	Class II-C	LOC_Os03g49880.1	Dispersed	Liu et al., 2019
	OsTCP8	Class II-B	LOC_Os03g57190.1	WGD or Segmental	Liu et al., 2019
	OsTCP9	Class I	LOC_Os04g11830.1	Dispersed	Liu et al., 2019
	OsTCP10	Class I	LOC_Os04g44440.1	WGD or Segmental	Liu et al., 2019
	OsTCP11	Class II-B	LOC_Os05g43760.1	WGD or Segmental	Liu et al., 2019
	OsTCP12	Class I	LOC_Os06g12230.1	WGD or Segmental	Liu et al., 2019
	OsTCP13	Class II-B	LOC_Os07g05720.1	WGD or Segmental	Liu et al., 2019
	OsTCP14	Class II-C	LOC_Os08g33530.1	WGD or Segmental	Liu et al., 2019
	OsTCP15	Class I	LOC_Os08g43160.1	WGD or Segmental	Liu et al., 2019
	OsTCP16	Class II-C	LOC_Os09g24480.1	WGD or Segmental	Liu et al., 2019
	OsTCP17	Class I	LOC_Os09g34950.1	WGD or Segmental	Liu et al., 2019
	OsTCP18	Class I	LOC_Os11g07460.1	WGD or Segmental	Liu et al., 2019
	OsTCP19	Class II-B	LOC_Os12g02090.1	Dispersed	Liu et al., 2019
	OsTCP20	Class I	LOC_Os12g07480.1	WGD or Segmental	Liu et al., 2019
	OsTCP21	Class II-B	LOC_Os12g42190.1	Dispersed	Liu et al., 2019
Populus	PtTCP1	Class I	Potri.001G060000.1		Liu et al., 2019
trichocarpa	PtTCP2	Class I	Potri.001G111800.1		Liu et al., 2019
	PtTCP3	Class I	Potri.001G327100.1		Liu et al., 2019
	PtTCP4	Class II-A	Potri.001G375800.1		Liu et al., 2019
	PtTCP5	Class I	Potri.002G152200.1		Liu et al., 2019
	PtTCP6	Class I	Potri.003G120200.1		Liu et al., 2019
	PtTCP7	Class I	Potri.003G167900.1		Liu et al., 2019
	PtTCP8	Class I	Potri.004G046300.1		Liu et al., 2019
	PtTCP9	Class II-B	Potri.004G065800.1		Liu et al., 2019
	PtTCP10	Class II-B	Potri.004G116100.1; Potri.T146100.1		Liu et al., 2019
	PtTCP11	Class I	Potri.004G222100.1		Liu et al., 2019
	PtTCP12	Class I	Potri.005G090300.1		Liu et al., 2019
	PtTCP13	Class I	Potri.006G125800.1; Potri.005G140600.1		Liu et al., 2019
	PtTCP14	Class II-C	Potri.008G115800.1		Liu et al., 2019
	PtTCP15	Class I	Potri.009G009400.1		Liu et al., 2019
	PtTCP16	Class II-C	Potri.010G130200.1		Liu et al., 2019
	PtTCP17	Class I	Potri.011G055500.1		Liu et al., 2019
	PtTCP18	Class II-B	Potri.011G083100.1		Liu et al., 2019
	PtTCP19	Class II-A	Potri.011G096600.1		Liu et al., 2019

	PtTCP20	Class II-C	Potri.012G059900.1	Liu et al., 2019
	PtTCP21	Class I	Potri.012G135900.1	Liu et al., 2019
	PtTCP22	Class I	Potri.013G110700.1	Liu et al., 2019
	PtTCP23	Class II-A	Potri.013G119400.1	Liu et al., 2019
	PtTCP24	Class I	Potri.014G078500.1	Liu et al., 2019
	PtTCP25	Class II-C	Potri.015G050500.1	Liu et al., 2019
	PtTCP26	Class II-B	Potri.015G058800.1	Liu et al., 2019
	PtTCP27	Class I	Potri.015G138200.1	Liu et al., 2019
	PtTCP28	Class I	Potri.016G094800.1	Liu et al., 2019
	PtTCP29	Class II-B	Potri.017G094800.1; Potri.016G074200.1	Liu et al., 2019
	PtTCP30	Class II-C	Potri.017G112000.1	Liu et al., 2019
	PtTCP31	Class I	Potri.019G081800.1	Liu et al., 2019
	PtTCP32	Class II-A	Potri.019G091300.1	Liu et al., 2019
	PtTCP33	Class I	Potri.T044100.1	Liu et al., 2019
Solanum lycopersicum	SlyTCP1	Class I	Solyc00g084870.2.1	Liu et al., 2019
	SlyTCP2	Class II-B	Solyc00g217560.1.1	Liu et al., 2019
	SlyTCP3	Class I	Solyc01g008230.2.1	Liu et al., 2019
	SlyTCP4	Class I	Solyc01g103780.2.1	Liu et al., 2019
	SlyTCP5	Class II-B	Solyc02g065800.1.1	Liu et al., 2019
	SlyTCP6	Class I	Solyc02g068200.1.1	Liu et al., 2019
	SlyTCP7	Class II-A	Solyc02g077250.2.1	Liu et al., 2019
	SlyTCP8	Class II-B	Solyc02g089020.1.1	Liu et al., 2019
	SlyTCP9	Class II-C	Solyc02g089830.1.1	Liu et al., 2019
	SlyTCP10	Class I	Solyc02g094290.1.1	Liu et al., 2019
	SlyTCP11	Class I	Solyc03g006800.1.1	Liu et al., 2019
	SlyTCP12	Class II-C	Solyc03g045030.1.1	Liu et al., 2019
	SlyTCP13	Class II-B	Solyc03g115010.1.1	Liu et al., 2019
	SlyTCP14	Class I	Solyc03g116320.2.1	Liu et al., 2019
	SlyTCP15	Class II-C	Solyc03g119770.2.1	Liu et al., 2019
	SlyTCP16	Class II-C	Solyc04g006980.1.1	Liu et al., 2019
	SlyTCP17	Class I	Solyc04g009180.1.1	Liu et al., 2019
	SlyTCP18	Class I	Solyc05g007420.1.1	Liu et al., 2019
	SlyTCP19	Class II-C	Solyc05g009900.1.1	Liu et al., 2019
	SlyTCP20	Class II-A	Solyc05g012840.1.1	Liu et al., 2019
	SlyTCP21	Class II-A	Solyc05g032780.1.1	Liu et al., 2019
	SlyTCP22	Class I	Solyc06g065190.1.1	Liu et al., 2019
	SlyTCP23	Class II-C	Solyc06g069240.1.1	Liu et al., 2019
	SlyTCP24	Class II-B	Solyc06g069460.1.1	Liu et al., 2019
	SlyTCP25	Class I	Solyc06g070900.2.1	Liu et al., 2019
	SlyTCP26	Class II-A	Solyc07g053410.2.1	Liu et al., 2019
	SlyTCP27	Class II-A	Solyc07g062680.1.1	Liu et al., 2019
	SlyTCP28	Class II-B	Solyc08g048370.2.1	Liu et al., 2019
	SlyTCP29	Class II-B	Solyc08g048390.1.1	Liu et al., 2019
	SlyTCP30	Class I	Solyc08g080150.1.1	Liu et al., 2019
	SlyTCP31	Class I	Solyc09g008030.1.1	Liu et al., 2019

		SlyTCP32	Class II-A	Solyc10g008780.1.1	Liu et al., 2019
		SlyTCP33	Class II-A	Solyc10g018710.1.1	Liu et al., 2019
		SlyTCP34	Class I	Solyc11g020670.1.1	Liu et al., 2019
		SlyTCP35	Class II-A	Solyc11g045640.1.1	Liu et al., 2019
		SlyTCP36	Class II-A	Solyc12g014140.1.1	Liu et al., 2019
	Zostera	ZmaTCP1	Class I	Zosma10g00260.1	Liu et al., 2019
	marina	ZmaTCP2	Class I	Zosma11g01120.1	Liu et al., 2019
		ZmaTCP3	Class II-C	Zosma135g00350.1	Liu et al., 2019
		ZmaTCP4	Class I	Zosma20g01250.1	Liu et al., 2019
		ZmaTCP5	Class II-C	Zosma220g00160.1	Liu et al., 2019
		ZmaTCP6	Class I	Zosma27g00610.1	Liu et al., 2019
		ZmaTCP7	Class II-A	Zosma343g00020.1	Liu et al., 2019
		ZmaTCP8	Class I	Zosma34g01230.1	Liu et al., 2019
		ZmaTCP9	Class II-A	Zosma40g00320.1	Liu et al., 2019
		ZmaTCP10	Class II-B	Zosma54g00550.1	Liu et al., 2019
		ZmaTCP11	Class II-A	Zosma78g00060.1	Liu et al., 2019
		ZmaTCP12	Class I	Zosma78g00840.1	Liu et al., 2019
		ZmaTCP13	Class I	Zosma89g00480.1	Liu et al., 2019
		ZmaTCP14	Class I	Zosma99g00440.1	Liu et al., 2019
**Gymnosperms**	*Amentotaxus*	AargTCP1	Class II-A	c52001_g1_i1	NCBI
	argotaenia	AargTCP2	Class II-A	c47794_g1_i4	NCBI
		AargTCP3	Class II-C	c46091_g1_i1	NCBI
		AargTCP4	Class II-B	c54022_g1_i1	NCBI
		AargTCP5	Class II-B	c53978_g1_i1	NCBI
		AargTCP6	Class I	c47631_g2_i1	NCBI
		AargTCP7	Class I	c50504_g1_i1	NCBI
		AargTCP8	Class I	c49922_g1_i1	NCBI
		AargTCP9	Class I	c54811_g2_i1	NCBI
		AargTCP10	Class I	c39031_g1_i1	NCBI
		AargTCP11	Class I	c44405_g1_i1	NCBI
		AargTCP12	Class I	c49698_g1_i1	NCBI
		AargTCP13	Class I	c52001_g2_i1	NCBI
		AargTCP14	Class II-A	c49546_g1_i1	NCBI
	Araucaria	AcunTCP1	Class II-A	c29690_g1_i1	NCBI
	cunninghamii	AcunTCP2	Class II-A	c49829_g1_i11	NCBI
		AcunTCP3	Class II-A	c49437_g2_i2	NCBI
		AcunTCP4	Class II-B	c44071_g1_i4	NCBI
		AcunTCP5	Class II-C	c37936_g1_i2	NCBI
		AcunTCP6	Class II-B	c45079_g1_i2	NCBI
		AcunTCP7	Class II-A	c46860_g3_i1	NCBI
		AcunTCP8	Class I	c26091_g2_i1+c26091_g1_i1	NCBI
		AcunTCP9	Class I	c34516_g1_i1	NCBI
		AcunTCP10	Class I	c32880_g1_i1	NCBI
		AcunTCP11	Class I	c67610_g1_i1	NCBI
		AcunTCP12	Class I	c38744_g1_i1	NCBI
		AcunTCP13	Class I	c39062_g2_i1	NCBI
		AcunTCP14	Class I	c67941_g1_i1	NCBI

	AcunTCP15	Class I	c25021_g1_i1		NCBI
Cephalotaxus sinensis	CepTCP1	Class II-C	c37352_g1_i2		NCBI
	CepTCP2	Class II-B	c41306_g1_i4		NCBI
	CepTCP3	Class II-A	c41854_g1_i2		NCBI
	CepTCP4	Class II-C	c12858_g1_i1		NCBI
	CepTCP5	Class II-A	c45353_g1_i5		NCBI
	CepTCP6	Class II-A	c41854_g2_i2		NCBI
	CepTCP7	Class II-C	c45012_g2_i1		NCBI
	CepTCP8	Class I	c32536_g1_i3		NCBI
	CepTCP9	Class I	c30511_g1_i1		NCBI
	CepTCP10	Class I	c29688_g1_i1		NCBI
	CepTCP11	Class I	c42990_g1_i1		NCBI
	CepTCP12	Class I	c40493_g1_i1		NCBI
	CepTCP13	Class I	c42559_g2_i1		NCBI
	CepTCP14	Class I	c48078_g2_i1		NCBI
Cunninghamia lanceolata	ClanTCP1	Class II-B	c18787_g1_i2		NCBI
	ClanTCP2	Class II-A	c33542_g1_i5		NCBI
	ClanTCP3	Class II-C	c21837_g1_i2		NCBI
	ClanTCP4	Class II-B	c23916_g1_i4		NCBI
	ClanTCP5	Class II-C	c25628_g1_i1		NCBI
	ClanTCP6	Class I	c20743_g1_i1		NCBI
	ClanTCP7	Class I	c30598_g1_i1		NCBI
	ClanTCP8	Class I	c31149_g2_i1		NCBI
	ClanTCP9	Class I	c30693_g2_i1		NCBI
	ClanTCP10	Class I	c35304_g4_i1		NCBI
	ClanTCP11	Class I	c32877_g2_i1		NCBI
	ClanTCP12	Class I	c35928_g3_i1		NCBI
Cycas panzhihuaensis	CpaTCP1	Class II-B	CYCAS_003196	Dispersed	CNGB
	CpaTCP2	Class II-A	CYCAS_003297	Dispersed	CNGB
	CpaTCP3	Class II-A	CYCAS_014483	Dispersed	CNGB
	CpaTCP4	Class II-B	CYCAS_018390	Dispersed	CNGB
	CpaTCP5	Class II-B	CYCAS_018393	Dispersed	CNGB
	CpaTCP6	Class II-A	CYCAS_028954	Dispersed	CNGB
	CpaTCP7	Class II-C	CYCAS_031463	Tandem	CNGB
	CpaTCP8	Class II-C	CYCAS_031464	Tandem	CNGB
	CpaTCP9	Class II-C	CYCAS_031466	Proximal	CNGB
	CpaTCP10	Class II-C	CYCAS_031467	Singleton	CNGB
	CpaTCP11	Class I	CYCAS_006008	Proximal	CNGB
	CpaTCP12	Class I	CYCAS_011180	Dispersed	CNGB
	CpaTCP13	Class I	CYCAS_024232	Dispersed	CNGB
	CpaTCP14	Class I	CYCAS_031374	Dispersed	CNGB
Ephedra equisetina	EpheTCP1	Class II-A	c33600_g1_i1		NCBI
	EpheTCP2	Class II-C	c31519_g1_i1		NCBI
	EpheTCP3	Class II-B	c31388_g1_i2		NCBI
	EpheTCP4	Class II-B	c15486_g1_i1		NCBI
	EpheTCP5	Class I	c22623_g2_i1		NCBI

	EpheTCP6	Class I	c26283_g1_i1		NCBI
	EpheTCP7	Class I	c22292_g1_i1		NCBI
	EpheTCP8	Class I	c4567_g1_i1		NCBI
	EpheTCP9	Class I	c27029_g1_i1		NCBI
	EpheTCP10	Class II-B	c26421_g2_i1		NCBI
Fokienia	FhodTCP1	Class II-B	c27120_g3_i1		NCBI
hodginsii	FhodTCP2	Class II-C	c5088_g1_i1		NCBI
	FhodTCP3	Class II-B	c37902_g1_i1		NCBI
	FhodTCP4	Class II-A	c33810_g1_i1		NCBI
	FhodTCP5	Class II-A	c37801_g1_i1		NCBI
	FhodTCP6	Class II-C	c30205_g1_i2		NCBI
	FhodTCP7	Class II-C	c15133_g1_i2		NCBI
	FhodTCP8	Class II-A	c22781_g1_i2		NCBI
	FhodTCP9	Class I	c32041_g1_i3		NCBI
	FhodTCP11	Class I	c29773_g1_i1		NCBI
	FhodTCP12	Class I	c38342_g1_i1		NCBI
	FhodTCP13	Class I	c33608_g2_i1		NCBI
	FhodTCP14	Class I	c34576_g1_i1		NCBI
	FhodTCP15	Class I	c22016_g1_i1		NCBI
	FhodTCP16	Class I	c28915_g1_i1		NCBI
Ginkgo	GbiTCP1	Class II-B	Gb_07891	Dispersed	NGDC
biloba	GbiTCP2	Class I	Gb_17362	Dispersed	NGDC
	GbiTCP3	Class I	Gb_17445	Dispersed	NGDC
	GbiTCP4	Class II-A	Gb_17863	Dispersed	NGDC
	GbiTCP5	Class II-A	Gb_18221	Dispersed	NGDC
	GbiTCP6	Class I	Gb_23937/Gb23938	Dispersed	NGDC
	GbiTCP7	Class I	Gb_24346	Dispersed	NGDC
	GbiTCP8	Class I	Gb_29528	Dispersed	NGDC
	GbiTCP9	Class I	Gb_30049	Proximal	NGDC
	GbiTCP10	Class II-C	Gb_34229	Dispersed	NGDC
	GbiTCP11	Class I	Gb_35810	Singleton	NGDC
	GbiTCP12	Class II-C	Gb_36354	Proximal	NGDC
	GbiTCP13	Class II-C	Gb_37187	Dispersed	NGDC
	GbiTCP14	Class II-A	Gb_38376	Tandem	NGDC
	GbiTCP15	Class II-C	Gb_38377	Tandem	NGDC
	GbiTCP16	Class II-C	Gb_40698	Singleton	NGDC
Gnetum	GmmTCP1	Class I	TnS000069033t01		NCBI
montanum	GmmTCP2	Class I	TnS000090013t03		NCBI
	GmmTCP3	Class II-B	TnS000208401t01		NCBI
	GmmTCP4	Class II-C	TnS000432977t01		NCBI
	GmmTCP5	Class II-C	TnS000432977t04		NCBI
	GmmTCP6	Class I	TnS000473551t05		NCBI
	GmmTCP7	Class II-B	TnS000547309t01		NCBI
	GmmTCP8	Class II-B	TnS000612329t03		NCBI
	GmmTCP9	Class II-C	TnS000831451t03		NCBI
	GmmTCP10	Class I	TnS000845445t02		NCBI
	GmmTCP11	Class II-A	TnS000898189t01		NCBI

	GmmTCP12	Class I	TnS001041269t11	NCBI
Metasequoia glyptostroboide	MetTCP1	Class II-A	c52624_g3_i1	NCBI
	MetTCP2	Class II-B	c47777_g1_i3	NCBI
	MetTCP3	Class II-B	c23714_g1_i1	NCBI
	MetTCP4	Class II-A	c46846_g1_i2	NCBI
	MetTCP5	Class II-C	c49302_g1_i1	NCBI
	MetTCP6	Class II-C	c42213_g1_i3	NCBI
	MetTCP7	Class II-C	c43902_g1_i1	NCBI
	MetTCP8	Class I	c35423_g1_i3	NCBI
	MetTCP9	Class I	c38117_g2_i1	NCBI
	MetTCP10	Class I	c51920_g1_i1	NCBI
	MetTCP11	Class I	c32918_g2_i1	NCBI
	MetTCP12	Class I	c57293_g4_i1	NCBI
	MetTCP13	Class I	c53306_g1_i1	NCBI
	MetTCP14	Class I	c25416_g1_i1	NCBI
Picea abies	PabTCP1	Class II-B	MA_101752g0020	Plantgenie
	PabTCP2	Class I	MA_102718g0020	Plantgenie
	PabTCP3	Class I	MA_10318773g0010	Plantgenie
	PabTCP4	Class II-B	MA_10426407g0020	Plantgenie
	PabTCP5	Class II-B	MA_12784g0010	Plantgenie
	PabTCP6	Class II-A	MA_1379g0010	Plantgenie
	PabTCP7	Class II-B	MA_15254g0010	Plantgenie
	PabTCP8	Class II-C	MA_16704g0010	Plantgenie
	PabTCP9	Class I	MA_16752g0010	Plantgenie
	PabTCP10	Class II-A	MA_178227g0010	Plantgenie
	PabTCP11	Class II-B	MA_189502g0010	Plantgenie
	PabTCP12	Class I	MA_2101g0010	Plantgenie
	PabTCP13	Class II-C	MA_35349g0010	Plantgenie
	PabTCP14	Class II-B	MA_37369g0010	Plantgenie
	PabTCP15	Class II-B	MA_3891g0010	Plantgenie
	PabTCP16	Class II-B	MA_4774g0010	Plantgenie
	PabTCP17	Class II-A	MA_52440g0010	Plantgenie
	PabTCP18	Class II-B	MA_547683g0010	Plantgenie
	PabTCP19	Class I	MA_635g0010	Plantgenie
	PabTCP20	Class II-C	MA_75780g0010	Plantgenie
	PabTCP21	Class I	MA_762628g0010	Plantgenie
	PabTCP22	Class I	MA_81710g0010	Plantgenie
	PabTCP23	Class II-C	MA_92659g0010	Plantgenie
	PabTCP24	Class II-B	MA_93117g0010	Plantgenie
Pinus lambertiana	PilaTCP1	Class I	PILA_05950	TreeGenes
	PilaTCP2	Class I	PILA_08502	TreeGenes
	PilaTCP3	Class I	PILA_14504	TreeGenes
	PilaTCP4	Class II-A	PILA_15258	TreeGenes
	PilaTCP6	Class II-C	PILA_18466	TreeGenes
	PilaTCP7	Class I	PILA_23482	TreeGenes
	PilaTCP9	Class I	PILA_27825	TreeGenes

	PilaTCP10	Class II-B	PILA_28581		TreeGenes
	PilaTCP11	Class I	PILA_28722		TreeGenes
	PilaTCP12	Class I	PILA_30758/PILA_16840		TreeGenes
	PilaTCP13	Class II-B	PILA_35774		TreeGenes
	PilaTCP14	Class II-A	PILA_36259		TreeGenes
	PilaTCP15	Class II-C	PILA_37973		TreeGenes
	PilaTCP16	Class I	PILA_37998		TreeGenes
Pinus	PtaTCP1	Class II-A	Pt0G22490.1	Dispersed	CNGB
tabuliformis	PtaTCP2	Class II-A	Pt0G35910.1	Dispersed	CNGB
	PtaTCP3	Class II-A	Pt2G12530.1	Dispersed	CNGB
	PtaTCP4	Class II-C	Pt2G34160.1	Proximal	CNGB
	PtaTCP5	Class II-C	Pt2G34180.1	Proximal	CNGB
	PtaTCP6	Class II-A	Pt3G48910.1	Dispersed	CNGB
	PtaTCP7	Class II-A	Pt4G23150.1	Dispersed	CNGB
	PtaTCP8	Class II-C	Pt5G21260.1	Proximal	CNGB
	PtaTCP9	Class II-C	Pt5G21330.1	Proximal	CNGB
	PtaTCP10	Class II-A	Pt5G59850.1	Proximal	CNGB
	PtaTCP11	Class II-B	Pt6G43400.1	Proximal	CNGB
	PtaTCP12	Class II-B	Pt6G43440.1	Proximal	CNGB
	PtaTCP13	Class II-B	Pt6G43460.1	Proximal	CNGB
	PtaTCP14	Class II-B	Pt6G43480.1	Tandem	CNGB
	PtaTCP15	Class II-B	Pt6G43490.1	Tandem	CNGB
	PtaTCP16	Class II-A	Pt9G27160.1	Dispersed	CNGB
	PtaTCP17	Class II-B	PtJG17690.1	Dispersed	CNGB
	PtaTCP18	Class II-A	PtJG45360.1	Dispersed	CNGB
	PtaTCP19	Class II-A	PtXG28290.1	Dispersed	CNGB
	PtaTCP20	Class II-A	PtXG33310.1	Proximal	CNGB
	PtaTCP21	Class I	Pt1G10100.1	Dispersed	CNGB
	PtaTCP22	Class I	Pt1G79940.1	Dispersed	CNGB
	PtaTCP23	Class I	Pt5G24770.1	Dispersed	CNGB
	PtaTCP24	Class I	Pt6G34430.1	Dispersed	CNGB
	PtaTCP25	Class I	Pt9G18760.1	Dispersed	CNGB
	PtaTCP26	Class I	Pt9G43570.1	Dispersed	CNGB
	PtaTCP27	Class I	PtQG06090.1	Dispersed	CNGB
	PtaTCP28	Class I	PtQG11760.1	Dispersed	CNGB
	PtaTCP29	Class I	PtXG25900.1	Dispersed	CNGB
Pinus taeda	PitaTCP1	Class II-B	PITA_09530		Plantgenie
	PitaTCP2	Class II-B	PITA_12692		Plantgenie
	PitaTCP3	Class II-B	PITA_15014		Plantgenie
	PitaTCP4	Class II-B	PITA_15274		Plantgenie
	PitaTCP5	Class II-B	PITA_17789		Plantgenie
	PitaTCP6	Class I	PITA_21443		Plantgenie
	PitaTCP7	Class II-B	PITA_24959		Plantgenie
	PitaTCP8	Class I	PITA_25541		Plantgenie
	PitaTCP9	Class I	PITA_25700		Plantgenie
	PitaTCP10	Class I	PITA_27053		Plantgenie
	PitaTCP12	Class II-C	PITA_35734		Plantgenie

	PitaTCP13	Class I	PITA_39176	Plantgenie
	PitaTCP14	Class I	PITA_41031	Plantgenie
	PitaTCP15	Class II-B	PITA_44314	Plantgenie
Platycladus	PoriTCP1	Class II-B	c36370_g2_i2	NCBI
orientalis	PoriTCP2	Class II-C	c22612_g1_i1	NCBI
	PoriTCP3	Class II-B	c35100_g1_i3	NCBI
	PoriTCP4	Class II-A	c36183_g1_i2	NCBI
	PoriTCP5	Class II-C	c34278_g2_i2	NCBI
	PoriTCP6	Class II-A	c41744_g1_i3	NCBI
	PoriTCP7	Class I	c21343_g1_i1	NCBI
	PoriTCP9	Class I	c19682_g1_i1	NCBI
	PoriTCP10	Class I	c29412_g1_i1	NCBI
	PoriTCP11	Class I	c38499_g2_i2	NCBI
	PoriTCP12	Class I	c19045_g2_i1	NCBI
	PoriTCP13	Class I	c36485_g1_i2	NCBI
	PoriTCP14	Class I	c39429_g1_i1	NCBI
Podocarpus	PmacTCP1	Class II-B	c37936_g1_i1	NCBI
macrophyllus	PmacTCP2	Class II-B	c36042_g1_i1	NCBI
	PmacTCP3	Class II-A	c49163_g1_i3	NCBI
	PmacTCP4	Class II-C	c19462_g1_i1	NCBI
	PmacTCP5	Class II-A	c56893_g1_i1	NCBI
	PmacTCP6	Class II-C	c27773_g2_i1	NCBI
	PmacTCP7	Class I	c50325_g1_i2	NCBI
	PmacTCP8	Class I	c31622_g1_i1	NCBI
	PmacTCP9	Class I	c37195_g1_i1	NCBI
	PmacTCP10	Class I	c47447_g1_i1	NCBI
	PmacTCP11	Class I	c1092_g1_i1	NCBI
	PmacTCP12	Class I	c48019_g3_i1	NCBI
	PmacTCP13	Class I	c4969_g1_i1	NCBI
	PmacTCP14	Class I	c41439_g1_i1	NCBI
Pseudotaxus	PchiTCP1	Class II-B	c48609_g1_i1	NCBI
chienii	PchiTCP2	Class II-A	c46127_g1_i5	NCBI
	PchiTCP3	Class I	c25835_g2_i1	NCBI
	PchiTCP4	Class I	c36731_g3_i1	NCBI
	PchiTCP5	Class I	c44338_g1_i1	NCBI
	PchiTCP6	Class I	c18850_g1_i1	NCBI
	PchiTCP7	Class I	c47354_g1_i1	NCBI
	PchiTCP8	Class I	c50182_g1_i1	NCBI
	PchiTCP9	Class I	c31824_g1_i1	NCBI
Pseudotsuga	PsmeTCP1	Class II-A	PSME_00771	TreeGenes
menziesii	PsmeTCP2	Class II-B	PSME_01457	TreeGenes
	PsmeTCP3	Class I	PSME_11161	TreeGenes
	PsmeTCP4	Class I	PSME_11162	TreeGenes
	PsmeTCP5	Class I	PSME_12280	TreeGenes
	PsmeTCP6	Class I	PSME_12667	TreeGenes
	PsmeTCP7	Class II-B	PSME_14975	TreeGenes

	PsmeTCP8	Class II-B	PSME_17261	TreeGenes
	PsmeTCP9	Class II-B	PSME_18749	TreeGenes
	PsmeTCP10	Class II-B	PSME_20418	TreeGenes
	PsmeTCP11	Class I	PSME_22519	TreeGenes
	PsmeTCP12	Class I	PSME_25171	TreeGenes
	PsmeTCP13	Class I	PSME_26507	TreeGenes
	PsmeTCP14	Class I	PSME_27453	TreeGenes
	PsmeTCP15	Class II-C	PSME_33238	TreeGenes
	PsmeTCP16	Class I	PSME_37590	TreeGenes
	PsmeTCP17	Class I	PSME_41354	TreeGenes
Sciadopitys	SverTCP1	Class II-A	c38858_g3_i2	NCBI
verticillata	SverTCP2	Class II-C	c22687_g1_i1	NCBI
	SverTCP3	Class II-B	c30217_g2_i1	NCBI
	SverTCP4	Class II-A	c36485_g1_i1	NCBI
	SverTCP5	Class II-B	c35001_g2_i2	NCBI
	SverTCP6	Class I	c30211_g1_i1	NCBI
	SverTCP7	Class I	c30831_g3_i1	NCBI
	SverTCP8	Class I	c33850_g1_i1	NCBI
	SverTCP9	Class I	c34460_g1_i2	NCBI
	SverTCP10	Class I	c41998_g1_i1	NCBI
	SverTCP11	Class I	c26081_g1_i1	NCBI
	SverTCP12	Class I	c30831_g1_i1	NCBI
Taxus	TwaTCP1	Class I	hds144570.1	CNGB
wallichiana	TwaTCP2	Class I	hds269320.1	CNGB
	TwaTCP3	Class I	hds311110.1	CNGB
	TwaTCP4	Class I	hds359070.1	CNGB
	TwaTCP5	Class I	hds365250.1	CNGB
	TwaTCP6	Class I	hds426070.1	CNGB
	TwaTCP7	Class II-A	hds015070.1	CNGB
	TwaTCP8	Class II-A	hds027250.1	CNGB
	TwaTCP9	Class II-C	hds080660.1	CNGB
	TwaTCP10	Class II-C	hds080680.1	CNGB
	TwaTCP11	Class II-A	hds158840.1	CNGB
	TwaTCP12	Class II-A	hds106850.1	CNGB
	TwaTCP13	Class II-C	hds183860.1	CNGB
	TwaTCP14	Class II-B	hds207430.1	CNGB
	TwaTCP15	Class II-A	hds273290.1	CNGB
	TwaTCP16	Class II-A	hds273310.1	CNGB
	TwaTCP17	Class II-A	hds273340.1	CNGB
	TwaTCP18	Class II-C	hds385570.1	CNGB
	TwaTCP19	Class II-C	hds385590.1	CNGB
	TwaTCP20	Class II-C	hds385640.1	CNGB
	TwaTCP21	Class II-C	hds385660.1	CNGB
	TwaTCP22	Class II-C	hds003850.1;hds003870.1	CNGB
	TwaTCP23	Class II-C	hds003910.1;hds003880.1	CNGB
	TwaTCP24	Class II-C	hds003890.1;hds281420.1	CNGB
Torreya	TjacTCP1	Class II-A	c24266_g1_i3	NCBI

	jackii	TjacTCP2	Class II-C	c25126_g2_i1		NCBI
		TjacTCP3	Class II-C	c10183_g1_i1		NCBI
		TjacTCP4	Class II-B	c27877_g1_i1		NCBI
		TjacTCP5	Class II-B	c19997_g1_i2		NCBI
		TjacTCP6	Class I	c14736_g1_i2		NCBI
		TjacTCP7	Class I	c38557_g1_i1		NCBI
		TjacTCP8	Class I	c21900_g1_i1		NCBI
		TjacTCP9	Class I	c27464_g1_i1		NCBI
		TjacTCP10	Class I	c18116_g1_i1		NCBI
		TjacTCP11	Class I	c22530_g1_i1		NCBI
		TjacTCP12	Class I	c17612_g1_i1		NCBI
		TjacTCP13	Class I	c21983_g3_i1		NCBI
	*Welwitschia_*	WelTCP1	Class II-B	c40719_g1_i1		CNGB
	mirabilis	WelTCP2	Class II-A	c8385_g1_i1		CNGB
		WelTCP3	Class II-B	c22268_g1_i1		CNGB
		WelTCP4	Class II-B	c23049_g1_i1		CNGB
		WelTCP5	Class I	c13671_g1_i1		CNGB
		WelTCP6	Class I	c72434_g1_i1		CNGB
		WelTCP7	Class I	c58966_g1_i1		CNGB
		WelTCP8	Class I	c11489_g1_i1		CNGB
		WelTCP9	Class I	c39483_g1_i1		CNGB
		WelTCP10	Class I	c22523_g1_i1		CNGB
		WelTCP11	Class I	c25348_g1_i1		CNGB
		WelTCP12	Class I	c55375_g1_i1		CNGB
		WelTCP13	Class I	c33069_g1_i1		CNGB
	Zamia	ZfuTCP1	Class II-B	c31650_g2_i1		NCBI
	furfuracea	ZfuTCP2	Class II-B	c23506_g1_i2		NCBI
		ZfuTCP3	Class II-A	c5463_g1_i1		NCBI
		ZfuTCP4	Class II-A	c29260_g2_i1		NCBI
		ZfuTCP5	Class I	c20974_g2_i1		NCBI
		ZfuTCP6	Class I	c34827_g1_i1		NCBI
		ZfuTCP7	Class I	c21778_g1_i1		NCBI
		ZfuTCP8	Class I	c27963_g2_i1		NCBI
		ZfuTCP9	Class I	c30473_g1_i2		NCBI
**Ferns**	*Adiantum*	AcauTCP1	Class I	c20559_g1_i1		NCBI
	caudatum	AcauTCP2	Class I	c27163_g1_i1		NCBI
		AcauTCP3	Class I	c25461_g1_i1		NCBI
		AcauTCP4	Class II-A	c23707_g1_i1		NCBI
		AcauTCP5	Class II-A	c26134_g1_i1		NCBI
		AcauTCP6	Class II-A	c26719_g1_i1		NCBI
		AcauTCP7	Class II-A	c26719_g2_i1		NCBI
	Alsophila	AspTCP1	Class I	Aspi01Gene03180	WGD or Segmental	figshare
	spinulosa	AspTCP2	Class I	Aspi01Gene05022	Dispersed	figshare
		AspTCP3	Class I	Aspi01Gene05356	Dispersed	figshare
		AspTCP4	Class I	Aspi01Gene06134	WGD or Segmental	figshare
		AspTCP5	Class I	Aspi01Gene42326	WGD or Segmental	figshare

	AspTCP6	Class I	Aspi01Gene42669	Dispersed	figshare
	AspTCP7	Class I	Aspi01Gene43392	Dispersed	figshare
	AspTCP8	Class I	Aspi01Gene45519	Dispersed	figshare
	AspTCP9	Class I	Aspi01Gene52526	Dispersed	figshare
	AspTCP10	Class I	Aspi01Gene53912	WGD or Segmental	figshare
	AspTCP11	Class I	Aspi01Gene54856	WGD or Segmental	figshare
	AspTCP12	Class I	Aspi01Gene56690	Dispersed	figshare
	AspTCP13	Class I	Aspi01Gene61342	Dispersed	figshare
	AspTCP14	Class I	Aspi01Gene65051	Dispersed	figshare
	AspTCP15	Class I	Aspi01Gene72040	WGD or Segmental	figshare
	AspTCP16	Class II-A	Aspi01Gene02912	WGD or Segmental	figshare
	AspTCP17	Class II-A	Aspi01Gene02916	Proximal	figshare
	AspTCP18	Class II-A	Aspi01Gene02917	Proximal	figshare
	AspTCP19	Class II-A	Aspi01Gene02918	Proximal	figshare
	AspTCP20	Class II-A	Aspi01Gene15466	Dispersed	figshare
	AspTCP21	Class II-A	Aspi01Gene21942	Dispersed	figshare
	AspTCP22	Class II-A	Aspi01Gene27741	Dispersed	figshare
	AspTCP23	Class II-A	Aspi01Gene48962	Dispersed	figshare
	AspTCP24	Class II-A	Aspi01Gene50266	WGD or Segmental	figshare
	AspTCP25	Class II-A	Aspi01Gene52180	Dispersed	figshare
	AspTCP26	Class II-A	Aspi01Gene55158	WGD or Segmental	figshare
	AspTCP27	Class II-A	Aspi01Gene55173	WGD or Segmental	figshare
	AspTCP28	Class II-A	Aspi01Gene55175	Tandem	figshare
	AspTCP29	Class II-A	Aspi01Gene55176	Tandem	figshare
	AspTCP30	Class II-A	Aspi01Gene61163	Dispersed	figshare
	AspTCP31	Class II-A	Aspi01Gene62817	WGD or Segmental	figshare
	AspTCP32	Class II-A	Aspi01Gene64947	Dispersed	figshare
Azolla	AzfiTCP1	Class I	Azfi_s0013.g013244		Fernbase
filiculoides	AzfiTCP2	Class I	Azfi_s0006.g010337		Fernbase
	AzfiTCP3	Class I	Azfi_s0271.g061235		Fernbase
	AzfiTCP4	Class I	Azfi_s0049.g030896		Fernbase
	AzfiTCP5	Class I	Azfi_s0060.g034817		Fernbase
	AzfiTCP6	Class I	Azfi_s0090.g042743		Fernbase
	AzfiTCP7	Class II-A	Azfi_s0003.g007839		Fernbase
	AzfiTCP8	Class II-A	Azfi_s0538.g076302		Fernbase
	AzfiTCP9	Class II-A	Azfi_s0168.g054602		Fernbase
	AzfiTCP10	Class II-A	Azfi_s0010.g012107		Fernbase
Ceratopteris	CerTCP1	Class I	c31330_g1_i2		NCBI
richardii	CerTCP2	Class I	c14454_g1_i1		NCBI
	CerTCP3	Class I	c8785_g1_i1		NCBI
	CerTCP4	Class I	c36204_g1_i1		NCBI
	CerTCP5	Class I	c22730_g1_i3		NCBI
	CerTCP6	Class II-A	c44533_g1_i1		NCBI
Equisetum	EdifTCP1	Class I	c43032_g1_i1		NCBI
diffusum	EdifTCP2	Class I	c35982_g2_i1		NCBI
	EdifTCP3	Class I	c28619_g1_i1		NCBI
	EdifTCP4	Class I	c27342_g1_i1		NCBI

		EdifTCP5	Class I	c19803_g1_i2	NCBI
		EdifTCP6	Class I	c34719_g1_i2	NCBI
		EdifTCP7	Class II-A	c21281_g1_i1	NCBI
		EdifTCP8	Class II-A	c45960_g1_i1	NCBI
	Lygodium	LygTCP1	Class I	c49914_g1_i1	NCBI
	flexuosum	LygTCP2	Class I	c30145_g1_i1	NCBI
		LygTCP3	Class I	c18073_g1_i1	NCBI
		LygTCP4	Class I	c68230_g1_i1	NCBI
	Pteridium	PterTCP1	Class I	c48935_g1_i1	NCBI
	aquilinum	PterTCP2	Class I	c48660_g1_i4	NCBI
		PterTCP3	Class I	c56354_g1_i1	NCBI
		PterTCP4	Class I	c43790_g1_i1	NCBI
		PterTCP5	Class I	c20016_g1_i1	NCBI
		PterTCP6	Class II-A	c64838_g1_i4	NCBI
	Salvinia	SacuTCP1	Class I	Sacu_v1.1_s0044.g013125	Fernbase
	cucullata	SacuTCP2	Class I	Sacu_v1.1_s0069.g016510	Fernbase
		SacuTCP3	Class I	Sacu_v1.1_s0073.g017047	Fernbase
		SacuTCP4	Class I	Sacu_v1.1_s0175.g024570	Fernbase
		SacuTCP5	Class I	Sacu_v1.1_s0068_cds	Fernbase
		SacuTCP6	Class II-A	Sacu_v1.1_s0168.g024243	Fernbase
		SacuTCP7	Class II-A	Sacu_v1.1_s0012_cds	Fernbase
**Lycophytes**	*Isoetes*	IengTCP1	Class I	JAANSK010026223.1	NCBI
	engelmannii	IengTCP2	Class I	JAANSK010001084.1	NCBI
		IengTCP3	Class I	JAANSK010001178.1	NCBI
		IengTCP4	Class II-A	JAANSK010006223.1	NCBI
		IengTCP5	Class II-A	JAANSK010016223.1	NCBI
		IengTCP6	Class II-A	JAANSK010000263.1	Liu et al., 2019
	Selaginella	SmTCP1	Class II-A	28794	Liu et al., 2019
	moellendorffii	SmTCP2	Class II-A	29162	Liu et al., 2019
		SmTCP3	Class I	404061/438214	Liu et al., 2019
		SmTCP4	Class I	scaffold_45:798620..798841	Liu et al., 2019
		SmTCP5	Class I	441321	Liu et al., 2019
		SmTCP6	Class II-A	89227	Liu et al., 2019
**Bryophytes**	*Anthoceros*	AnagTCP1	Class I	VJWM01000108.1	NCBI
	angustus	AnagTCP2	Class II-A	VJWM01000192.1	NCBI
	Marchantia	MpTCP1	Class II-A	Mapoly0001s0298.1.p	Liu et al., 2019
	polymorpha	MpTCP2	Class I	Mapoly0068s0102.1.p	Liu et al., 2019
	Physcomitrella	PpTCP1	Class I	Pp3c10_14310V3.1.p	Liu et al., 2019
	patens	PpTCP2	Class I	Pp3c10_20400V3.1.p	Liu et al., 2019
		PpTCP3	Class II-A	Pp3c11_26670V3.1.p	Liu et al., 2019
		PpTCP4	Class I	Pp3c3_24450V3.1.p	Liu et al., 2019
		PpTCP5	Class I	Pp3c3_24660V3.1.p	Liu et al., 2019
		PpTCP7	Class II-A	Pp3c7_5230V3.2.p	Liu et al., 2019
	Sphagnum	SfaTCP1	Class I	Sphfalx0001s0381.1.p	Liu et al., 2019
	fallax	SfaTCP2	Class I	Sphfalx0023s0122.1.p	Liu et al., 2019
		SfaTCP3	Class I	Sphfalx0067s0096.1.p	Liu et al., 2019

		SfaTCP4	Class I	Sphfalx0188s0014.1.p	Liu et al., 2019
		SfaTCP5	Class I	Sphfalx0231s0014.1.p	Liu et al., 2019
**Algae**	*Mesotaenium*	MendTCP1	Class I	ME000298S05127/S298	figshare
	endlicherianum	MendTCP2	Class II-A	S210	figshare
	Spirogloea	SmusTCP1	Class I	SM000060S19623/s60	figshare
	muscicola	SmusTCP2	Class I	SM003533S13204/s3533	figshare
		SmusTCP3	Class I	SM000037S13520/s37	figshare
		SmusTCP4	Class II	s63	figshare
		SmusTCP5	Class II	s398	figshare
		SmusTCP6	Class II	s177	figshare

*Database websites: CNGB, https://db.cngb.org/; Fernbase, https://www.fernbase.org/; figshare, https://figshare.com; NCBI, https://www.ncbi.nlm.nih.gov/; NGDC, https://ngdc.cncb.ac.cn/; Phytozome, http://phytozome.jgi.doe.gov/; Plantgenie, https://plantgenie.org/; TreeGenes, https://treegenesdb.org/.

REFERENCES

Liu, M. M., Wang, M. M., Yang, J., Wen, J., Guo, P. C., Wu, Y. W., et al. (2019). Evolutionary and comparative expression analyses of TCP transcription factor gene family in land plants. *Int. J. Mol. Sci*. 20, 3591. doi: 10.3390/ijms20143591
