# Supplementary material for: Comprehensive evolutionary analysis of the TCP gene family: Further insights for its origin, expansion, and diversification
Source: Front Plant Sci. 2022 Sep 2;13:994567. doi: 10.3389/fpls.2022.994567 (PMC9480096; doi:10.3389/fpls.2022.994567)

Supplementary Figure 1

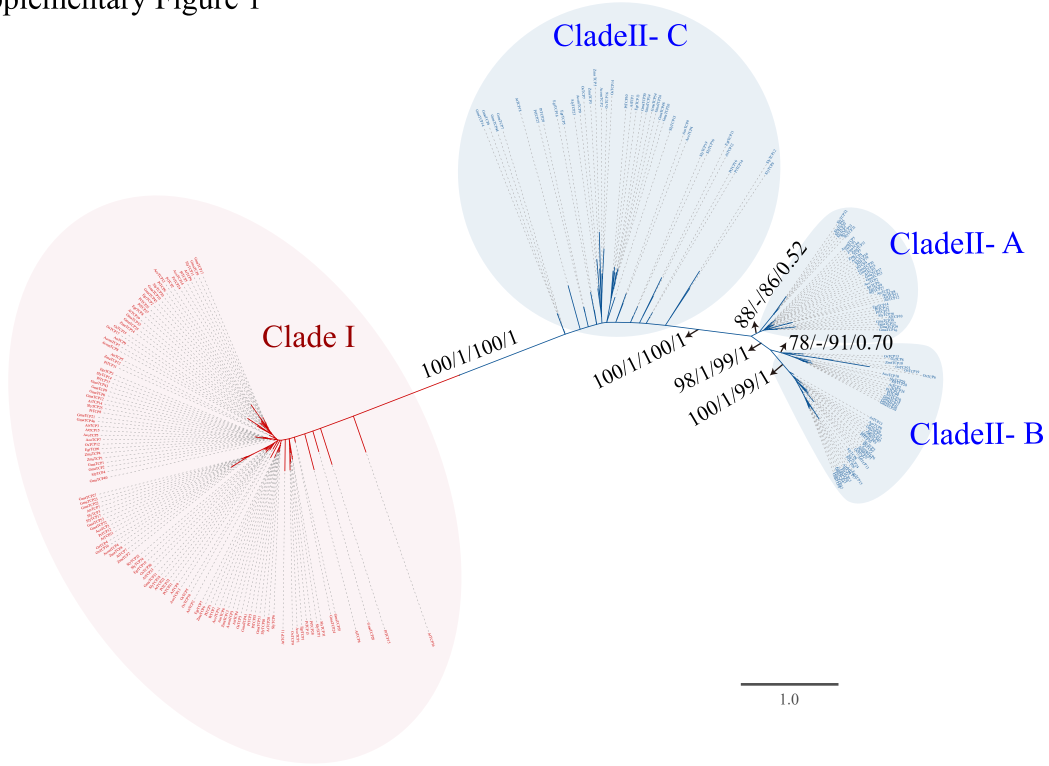

Supplementary Figure 2

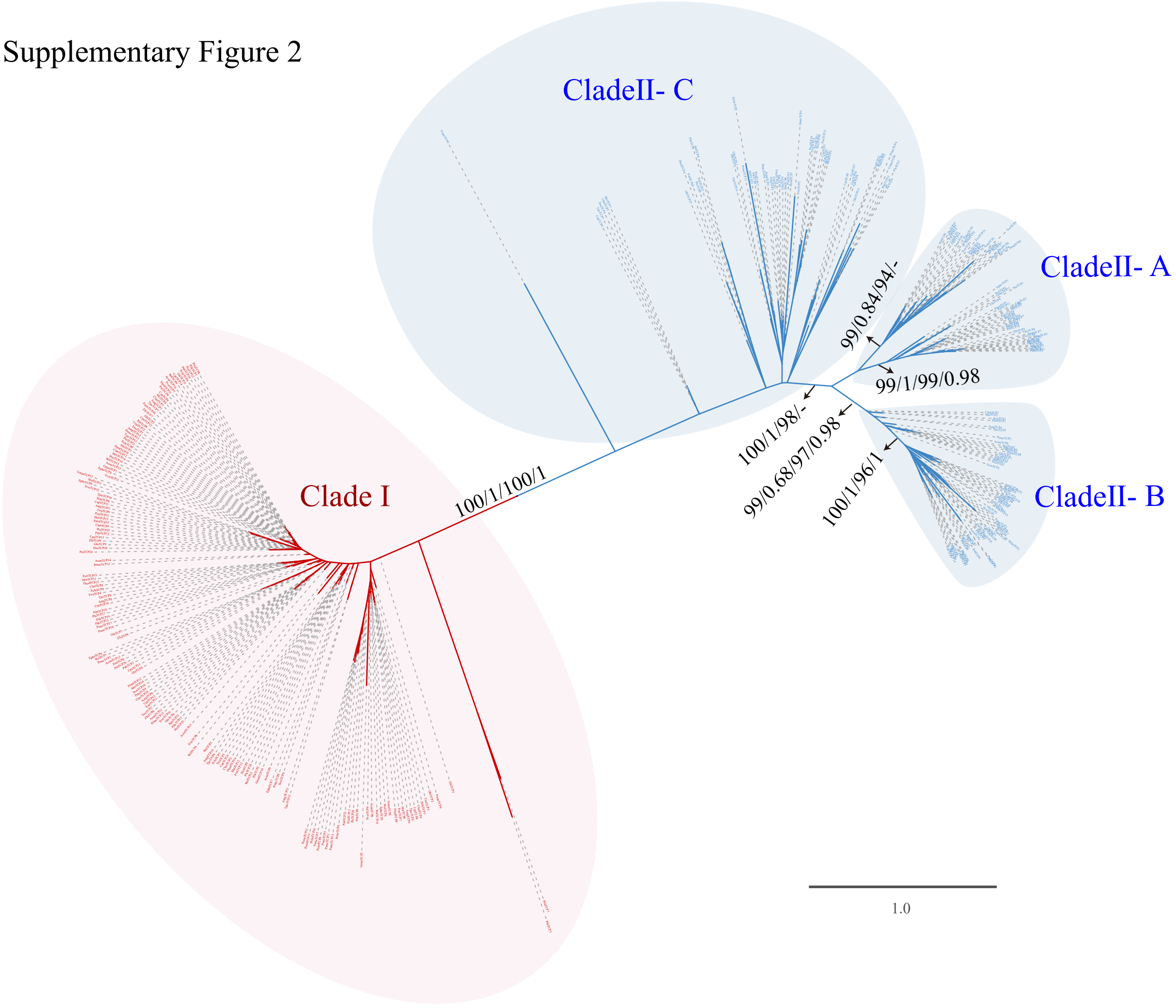

Supplementary Figure 3

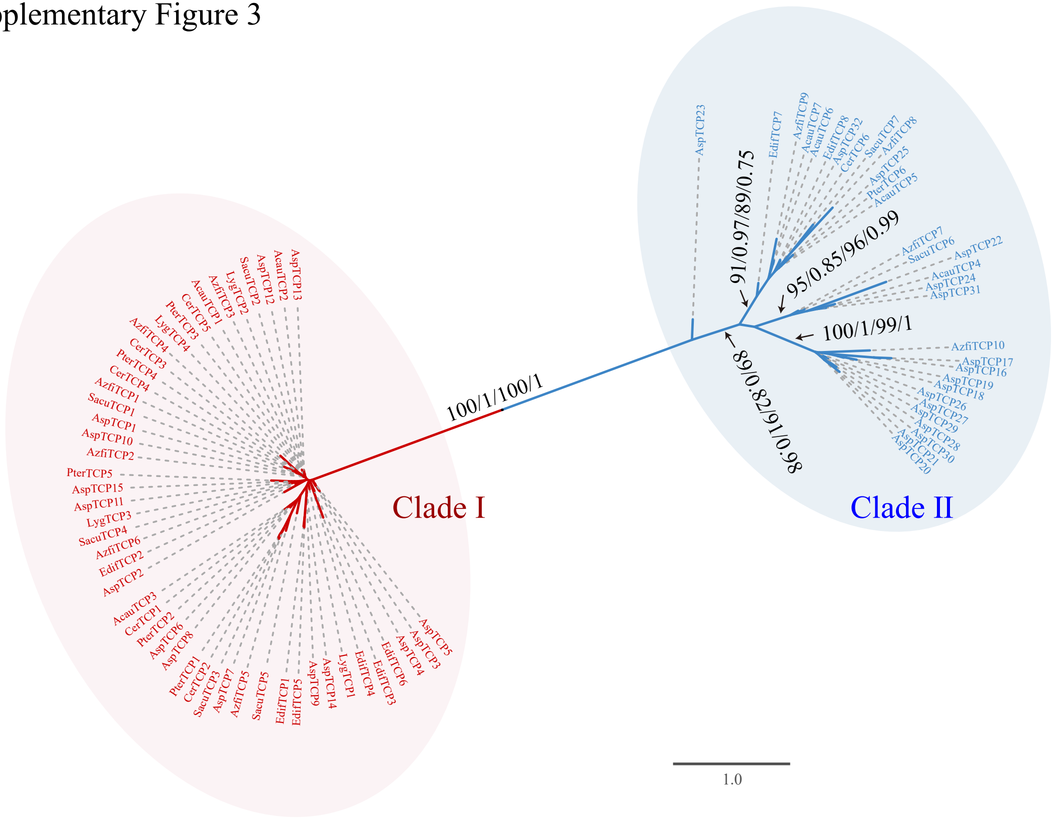

Supplementary Figure 4

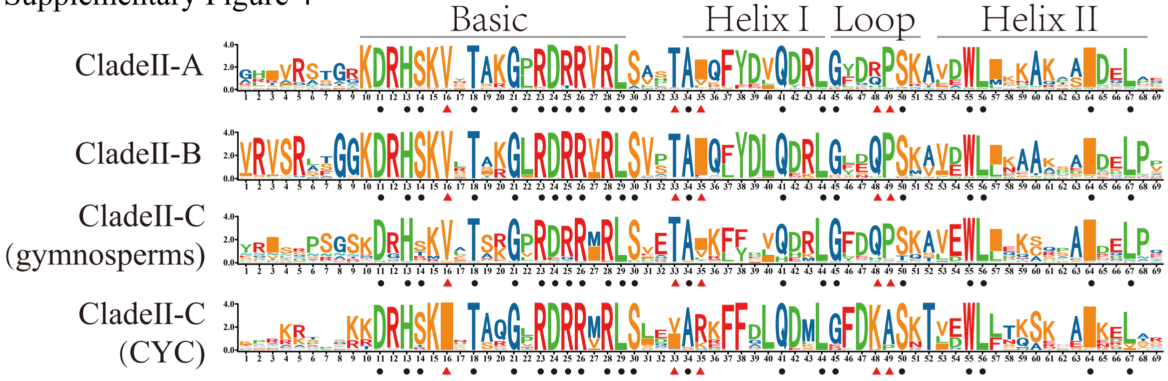

Supplementary Figure 5

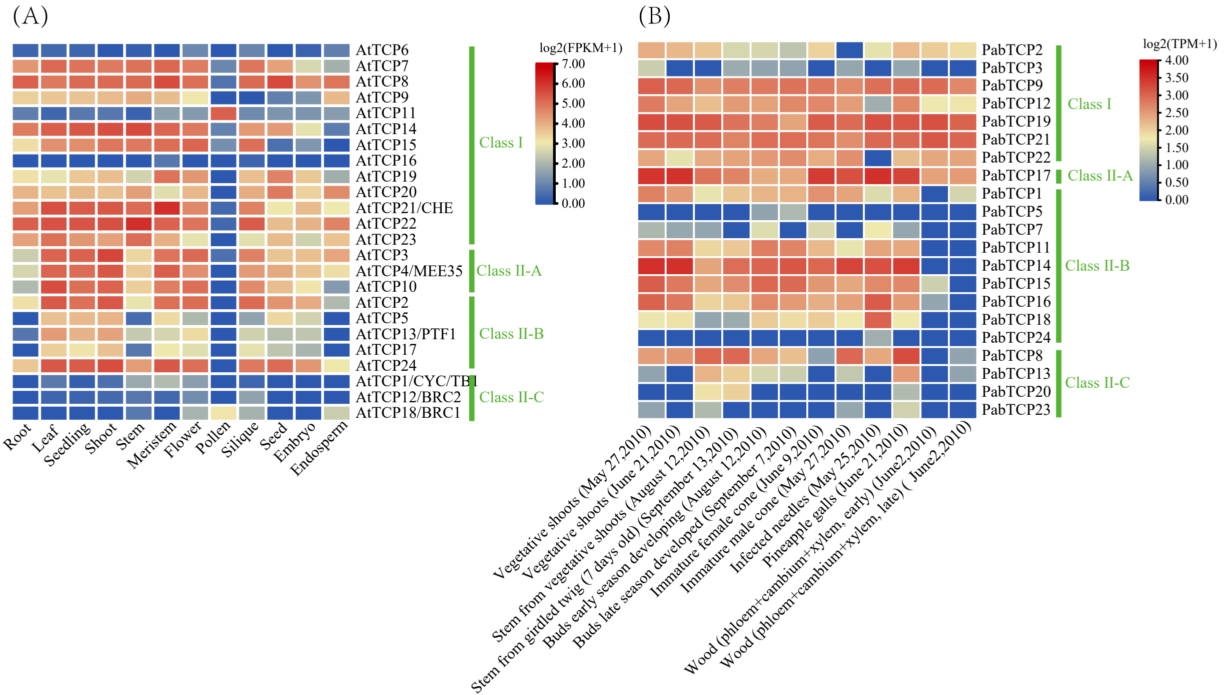

Supplement: Supplementary file 2 [file Data_Sheet_2.pdf]
